# Mechanistic roles of neutrophil heterogeneity in tumour pathogenesis

**DOI:** 10.3389/fimmu.2025.1721090

**Published:** 2025-12-10

**Authors:** Hua Lin, Yutian Liao, Zhonghui Chen, Ye Chen, Yating Chen, Yulin Wang, Zhanfei Chen

**Affiliations:** 1 The Affiliated Hospital of Putian University, Putian University, Putian, Fujian, China; 2 School of Basic Medical Sciences, Putian University, Putian, Fujian, China; 3 College of Pharmacy and Medical Technology, Putian University, Putian, Fujian, China

**Keywords:** neutrophils, heterogeneity, tumour, immune cells, therapy

## Abstract

Neutrophils are the body’s primary responders to infection and injury, yet they also exert diverse effects within tumours through distinct subtypes and mechanisms of action. In light of persistent challenges in clinical oncology, including drug resistance, a research focus on neutrophil biology represents a promising frontier. This review examines neutrophil heterogeneity in cancer by exploring their developmental stages, tumour-specific mechanisms influencing progression, and established classification systems. It further highlights emerging neutrophil subpopulations identified across specific tumours and disease contexts, offering insights into their dual roles in pathogenesis. By integrating recent findings, this work provides a framework to guide drug development and clinical therapeutics in oncology and related pathologies.

## 1 Introduction

Neutrophils, also known as polymorphonuclear neutrophils (PMNs), are the most abundant type of white blood cells in human circulation and a key component of the innate immune system (1). They play a crucial role in infections, tissue injury, and chronic diseases. In recent years, neutrophil heterogeneity has emerged as a growing research focus, with accumulating evidence showing that neutrophil populations display diverse functional properties under both homeostatic and pathological conditions (2). During neutrophil development, microenvironmental conditions can direct their differentiation into different subsets, challenging the traditional view of neutrophils as a homogeneous population. Owing to their broad and context-specific functions in innate immunity, neutrophils have also opened new perspectives in cancer therapy. Integrating neutrophil heterogeneity into tumour treatment represents a relatively novel research direction. Although promising advances have been achieved, a more precise understanding of how neutrophil heterogeneity specifically influences tumour progression is still needed for clinical translation.

Current research techniques enable relatively accurate classification of neutrophils based on surface-specific molecular structures, such as the human neutrophil antigen (HNA) system. The HNA system comprises polymorphic, neutrophil-specific surface antigens that participate in intercellular recognition and signal transduction. In 1998, the Granulocyte Antigen Working Group of the International Society of Blood Transfusion (ISBT) established a standardised nomenclature for well-defined neutrophil antigens based on their glycoprotein localisation, designating them as “human neutrophil alloantigen” (HNA) to reflect their expression on neutrophils (3). To date, the ISBT Granulocyte Immunobiology Working Party (GIWP) has identified five HNA antigen systems: HNA-1, HNA-2, HNA-3, HNA-4, and HNA-5 (4). Among these, two antigen groups (HNA-1 and HNA-2) are expressed exclusively on neutrophils and are sometimes referred to by their historical names (NA and NB, respectively), as they are truly neutrophil-specific and not shared with other primate cells (5). By contrast, other HNA antigens (e.g., HNA-3) exhibit broader tissue distribution, including expression on lymphocytes, platelets, and pulmonary endothelial cells (6). Nevertheless, due to their clinical relevance in neutrophil biology, these antigens have also been incorporated into the HNA system.

In this context, this article synthesises experimental studies and review literature to clarify neutrophil developmental processes and their cancer-associated heterogeneity in the setting of specific tumours, aiming to provide a consolidated perspective for future research.

## 2 Developmental stages of neutrophils

### 2.1 Circulatory system/blood

Neutrophils are derived from granulocyte-macrophage progenitors. Prior research has identified three distinct neutrophil subpopulations within human bone marrow: precursor neutrophils, non-proliferative neutrophils, and mature neutrophils. Neutrophil precursors undergo differentiation into both immature and mature neutrophils (7). Under physiological conditions, the development of neutrophils follows a sequential progression through several stages, including hematopoietic stem cells, multipotent progenitors, common myeloid progenitors, early unipotent neutrophil progenitors, pre-neutrophils, myelocytes, metamyelocytes, band cells, and mature neutrophils, each distinguished by specific surface markers. Within the tumour microenvironment (TME), neutrophils are typically classified into anti-tumour and pro-tumour subtypes (8).

**Figure 1:**
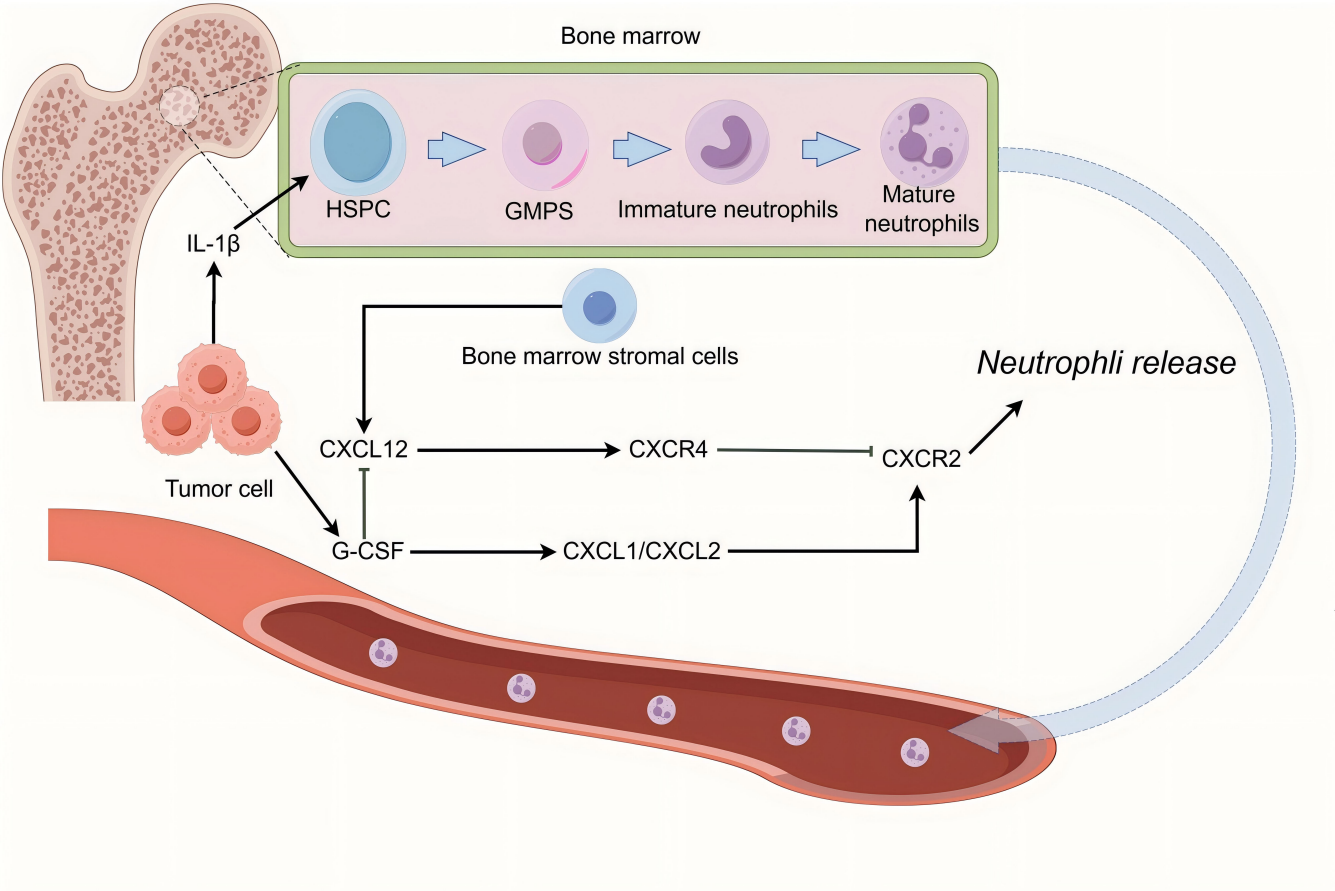
Tumour-induced mobilisation of neutrophils from haematopoiesis to the tumour microenvironment. Haematopoietic stem cells (HSCs) serve as progenitors of neutrophils during early development. HSCs give rise to granulocyte-macrophage progenitors (GMPs), which proliferate into hNePs before differentiating into mature neutrophils. These mature neutrophils reside in the bone marrow for 4–6 days before entering circulation. Their release is regulated by inflammatory chemokine receptors: C-X-C motif chemokine receptor (CXCR)2 promotes neutrophil mobilisation, whereas CXCR4 mediates neutrophil retention. Tumour cells secrete granulocyte colony-stimulating factor (G-CSF), which stimulates the production of C-X-C motif chemokine ligand (CXCL)1 and CXCL2. tumour-released proinflammatory substances like G-CSF and IL-1β signal the bone marrow, causing hematopoietic issues and continuous systemic neutrophil proliferation (9). G-CSF also facilitates mobilisation by downregulating CXCL12 expression in the bone marrow, thereby promoting the egress of immature neutrophils—cells with incomplete immune functions-into the bloodstream. In addition, tumour-derived interleukin-1β (IL-1β) enhances the expansion and development of HSCs within the bone marrow, accelerating neutrophil production and maturation. The mobilisation of neutrophils into circulation by tumour cells is a prerequisite for their infiltration into the TME, where they differentiate into tumour-associated neutrophil (TANs) (10, 11).

TANs exert profound effects on tumour biology and exhibit multiple subtypes (**Figure 1**). In healthy individuals, circulating neutrophils exist at different densities, with functional specialisation: low-density neutrophils (LDNs) display antimicrobial activity and lymphocyte suppression, whereas high-density neutrophils (HDNs) exhibit weaker effector functions. These subsets are rarely detected under physiological conditions (12). For decades, neutrophils were considered terminally differentiated cells restricted to antimicrobial defence and inflammatory responses (11, 12). This paradigm has been challenged by evidence demonstrating that LDNs and HDNs exert both tumour-promoting and tumour-suppressing effects. Using discontinuous density gradient separation, neutrophils were isolated from the high-density granulocyte fraction, whereas low-density (LD) monocytes were obtained from peripheral blood mononuclear cells (PBMCs). This methodology allowed researchers to classify circulating neutrophils into HDNs and LDNs. In cancer, LDNs exhibit both mature and immature morphological phenotypes, although the underlying mechanisms remain unclear. LDNs may also arise from the HD fraction, serving as a source of mature neutrophils. Notably, the spontaneous conversion of HDNs into LDNs has been observed in the circulation of late-stage tumour-bearing mice (13).

Cell surface marker profiling remains the most reliable approach for neutrophil subtyping. In cancer patients, circulating neutrophils include three granulocyte subsets: mature segmented HDNs, mature LDNs, and immature LDNs. Importantly, HDNs and LDNs are not homogeneous populations but represent subtypes defined by specific molecular signatures. Recent research has elucidated the complex roles of neutrophils in cancer, highlighting their involvement in tumour growth and metastasis, maintenance of cancer stem cells, regulation of cell cycle progression, impairment of immune surveillance, and co-modulation of T cell responses (14). These subsets exhibit distinct surface marker profiles and fulfil differential roles in tumour biology (**Table 1**).

**Table 1:** Tumour-modulating effects of neutrophil-associated molecules (14–16).

Related molecules	Roles	Molecular mechanism
ROS, RNS, Proteases	Tumour-promoting	Neutrophils induce epithelial damage and pro-tumourigenic inflammation through ROS/RNS and protease secretion, facilitating epithelial-to-cancer cell transformation (17).
TNF, IL-17, CD4^+^ T cells	Tumour-promoting	Neutrophils are activated by TNF-induced IL-17^+^CD4^+^ T lymphocytes.
IL-1R	Tumour-promoting	Neutrophils convert senescent cancer cells into proliferative cells via IL-1 receptor antagonist (IL-1Ra).
NE, IRS1, PI3K	Tumour-promoting	NE translocates to cancer cells, degrades IRS1, and activates PI3K signalling to directly stimulate proliferation.
ARG1	Immunosuppression	Arg-1-mediated L-arginine depletion suppresses CD8^+^ T cell function (18).
TGFβ	Immunosuppression	Neutrophils mediate immune suppression through TGFβ signalling.
CXCL1/2/5, Hypoxia	Tumour-suppressive	Hypoxia-induced CXCL1/2/5 secretion recruits tumouricidal neutrophils (19).
MET, TNF, inducible nitric oxide synthase (iNOS)	Tumour-suppressive	Endothelial MET receptor upregulation triggers TNF-dependent iNOS production in neutrophils, inducing cytotoxic effects on cancer cells.
BV8, MMP-9, VEGF-A	Pro-angiogenic	Neutrophil-derived BV8 and MMP-9 activate VEGF-A, remodelling ECM and inducing angiogenesis (15).
CD11b	Pro-metastatic	CD11b-dependent neutrophil guidance promotes cancer-endothelial cell co-localisation during early metastasis.
HMGB1, TLR4, IL-17, NETs, LTB4, iNOS	Dual roles (Promotion/Suppression)	Bidirectional regulation via: • Pro-metastatic: HMGB1-TLR4 signalling, IL-17^+^ γδT cell activation, NETosis, LTB4 • Antitumour: iNOS-mediated T cell suppression (15)

#### 2.1.1 LDN

In non-oncological contexts, LDNs were first described in 1986 in patients with systemic lupus erythematosus (SLE) and rheumatoid arthritis. In 2003, Bennett et al. performed microarray analysis of PBMCs from paediatric SLE patients and identified elevated expression of neutrophil-specific genes. This “granulocyte signature” was attributed to increased LDNs within the PBMC fraction (20). In the TME, neutrophils exhibit phenotypic plasticity, giving rise to a distinct LDN subpopulation. Tumour-associated LDNs emerge transiently during inflammation and progressively accumulate in malignancies. This subset consists of at least two morphologically different neutrophil populations regulated by discrete immunomodulatory mechanisms. In 4T1 mouse models, circulating neutrophils progressively increased during cancer progression. In healthy mice, HDNs accounted for >95% of total neutrophils. However, tumour growth was accompanied by an increase in LDNs and a proportional decrease in HDNs. Importantly, the rise in LDNs was not explained solely by HDN conversion, since the absolute HDN count remained stable, suggesting that most LDNs arise *de novo* during disease progression (12). Morphologically, LDNs share similar granularity with HDNs but are markedly larger. Spontaneous conversion of HDNs into LDNs was observed in late-stage tumour-bearing mice, occurring more frequently than the reverse process. This interconversion is mediated by transforming growth factor-β (TGF-β) (21). In humans, LDNs are elevated in the blood of breast cancer (BC) patients, particularly those with metastatic disease. Higher LDN prevalence has been associated with poor response to neoadjuvant chemotherapy (22, 23).

##### 2.1.1.1 Subpopulations and functions of LDNs

Mature LDNs originate from the bone marrow and spleen. Two major subpopulations, mature and immature, have been identified, both characterised by lower density in peripheral blood (21). LDNs are not restricted to pathological states and can also appear in healthy adults when peripheral neutrophils undergo activation-induced density changes. Functionally, LDNs foster a tumour-supportive microenvironment by suppressing cytotoxicity against tumour cells and strongly inhibiting cluster of differentiation (CD)8^+^ T-cell proliferation. They also exhibit a diminished inflammatory profile, with reduced expression of CXCL1, CXCL2, CXCL10, C-C motif chemokine ligand (CCL) 2, CCL3, C-C chemokine receptor type 5 (CCR5), CXCR2, and cluster of differentiation 62 ligand (CD62L). Compared with HDNs, mature human LDNs display higher levels of activation markers integrin alpha M (CD11b) and CD66b. Three neutrophil subpopulations have been described within the LDN fraction, with notable interindividual variability: CD16^+^(Fc gamma receptor III)/CD11b^+^, CD16^-^/CD11b^+^, and CD16^-^/CD11b^-^. Since CD11b and CD66b localise to secretory vesicles, gelatinase granules, and/or specific granules, their expression intensity likely reflects activation and degranulation states. LDNs exhibit reduced apoptosis, prolonging their lifespan (11). Moreover, HDNs that transition into LDNs acquire immunosuppressive functions. Clinically, elevated circulating LDNs correlate with poor therapeutic response and reduced survival (24). Key Functions of LDNs: Impaired phagocytosis, Reduced oxidative burst, Inhibition of CD8^+^ T cell proliferation, Prolonged neutrophil survival (11).

Immature LDNs (iLDNs) resemble polymorphonuclear myeloid-derived suppressor cells (PMN-MDSCs), although direct classification as PMN-MDSCs remains unproven. In patients with lung or ovarian cancer, two subsets have been identified: CD45high LDNs, which suppress T-cell proliferation and display mature morphology, and CD45low LDNs, which are immature and lack immunosuppressive activity (24). Mature LDNs inhibit immune-mediated tumour clearance and promote metastasis through cytokine and chemoattractant secretion, including CXCL2 and vascular endothelial growth factor (VEGF).

#### 2.1.2 HDNs

HDNs represent the predominant subset of mature neutrophils and are central to antitumour immunity. Characterised by segmented nuclei and higher density, they are typically isolated from the high-density fraction using gradient centrifugation. HDNs express mature neutrophil markers such as CD66b, CD11b, termed 3-fucosyl-N-acetyl-lactosamine (CD15), and CD16 in humans, or Lymphocyte Antigen 6 Complex, Locus G​ (Ly6G) in mice (11, 25). CD11b and CD66b also serve as activation markers. Within the TME, HDNs are regulated by TGF-β, Interleukin-8 (IL-8), and G-CSF, which can drive degranulation or dedifferentiation into LDNs. HDN phenotype shifts with tumour stage: in early disease, HDNs exhibit antitumour N1 characteristics, whereas in advanced tumours, TGF-β polarises them into a protumourigenic N2 phenotype, marked by secretion of pro-angiogenic and pro-metastatic factors such as VEGF and matrix metalloproteinase-9 (MMP-9) (11).

##### 2.1.2.1 Role of HDN

HDNs generally function as tumour suppressors with cytotoxic capabilities. They exert antitumour activity through production of reactive oxygen species (ROS) (26), release of proteases (e.g., myeloperoxidase [MPO]), and direct contact-mediated tumour cell killing. In early tumour stages, HDNs strongly inhibit tumour cell migration to pre-metastatic niches. Their Fc receptors (e.g., CD16) recognise tumour antigen-antibody complexes, mediating antibody-dependent cellular cytotoxicity (ADCC), exemplified by the antitumour effect of anti-human epidermal growth factor receptor 2 (HER2) antibodies in BC (11, 27). A subset of immunostimulatory HDNs also functions as antigen-presenting cells (APCs), activating T-cell proliferation via upregulation of costimulatory molecules such as CD86 and tumour necrosis factor ligand superfamily member 4 (OX40L). The HDN-to-LDN ratio, reflected in the neutrophil-to-lymphocyte ratio (NLR), serves as an independent prognostic marker in several cancers, including lung and BC (28). A higher proportion of HDNs generally predicts favourable outcomes. Therapeutically, strategies include enhancing HDN activity (e.g., with interferon-β [IFN-β] or TGF-β inhibitors), blocking their conversion to LDNs, and reversing immunosuppressive phenotypes using monoclonal antibodies such as programmed death-ligand 1 (PD-L1) inhibitors (11).

#### 2.1.3 PMN-MDSCs

Myeloid-derived suppressor cells (MDSCs) are a heterogeneous population of immunosuppressive myeloid cells that arise under pathological conditions from aberrantly activated bone marrow progenitors and immature myeloid cells. They reside in the bone marrow, peripheral blood, spleen, liver, lungs, and tumour tissues, and are broadly classified into PMN-MDSCs and monocytic myeloid-derived suppressor cells (M-MDSCs). MDSCs are defined by their expression of CD11b and granulocyte receptor-1 (Gr-1) (2), and their expansion is driven by dysregulated cytokine expression in cancer, infection, and inflammatory disorders. PMN-MDSCs, a granulocytic subset of MDSCs (29), display potent immunosuppressive activity within the TME. They inhibit antitumour immunity through multiple mechanisms: (i) expression of immune checkpoint ligands such as PD-L1, which engages programmed cell death protein 1 (PD-1) on T cells to induce exhaustion; (ii) secretion of immunosuppressive mediators including nitric oxide (NO), arginase-1 (Arg-1), and ROS, which impair T-cell proliferation and function; and (iii) recruitment of regulatory T cells (Tregs), thereby reinforcing an immunosuppressive milieu and suppressing effector T-cell responses (30).

PMN-MDSCs also suppress natural killer (NK) cell activity. By releasing NO and TGF-β, they impair NK cell cytotoxicity and activation, weakening their tumouricidal potential (2). In addition, PMN-MDSCs promote tumour progression by secreting VEGF to stimulate angiogenesis, releasing MMP-9 to remodel the extracellular matrix (ECM) and facilitate invasion, and contributing to the establishment of pre-metastatic niches in distant organs. They also induce epithelial-mesenchymal transition (EMT) in tumour cells, thereby enhancing invasiveness and metastatic potential (31).

Despite extensive research, it remains unclear whether circulating neutrophils and PMN-MDSCs represent the same population or distinct subsets (32, 33). Both share common surface markers and morphological features, leading to persistent controversy. LDNs and PMN-MDSCs also demonstrate phenotypic and density-related similarities (31, 34), further complicating their distinction. Currently, no definitive method exists to discriminate neutrophils from polymorphonuclear myeloid-derived suppressor cells (PMN-MDSCs), and whether they constitute separate entities remains unresolved.

### 2.2 Intratumoural neutrophils

During tumour progression, malignant cells undergo sequential stages, including formation and growth, detachment from the primary site, entry into the circulation, and colonisation of distant organs. Metastasis requires several key capacities: invasion (detachment from the primary tumour, penetration of the basement membrane, and infiltration of adjacent tissues, blood vessels, or lymphatics), extravasation (migration of tumour cells across vascular or lymphatic walls into new tissues), and colonisation at secondary sites.

**Figure 2:**
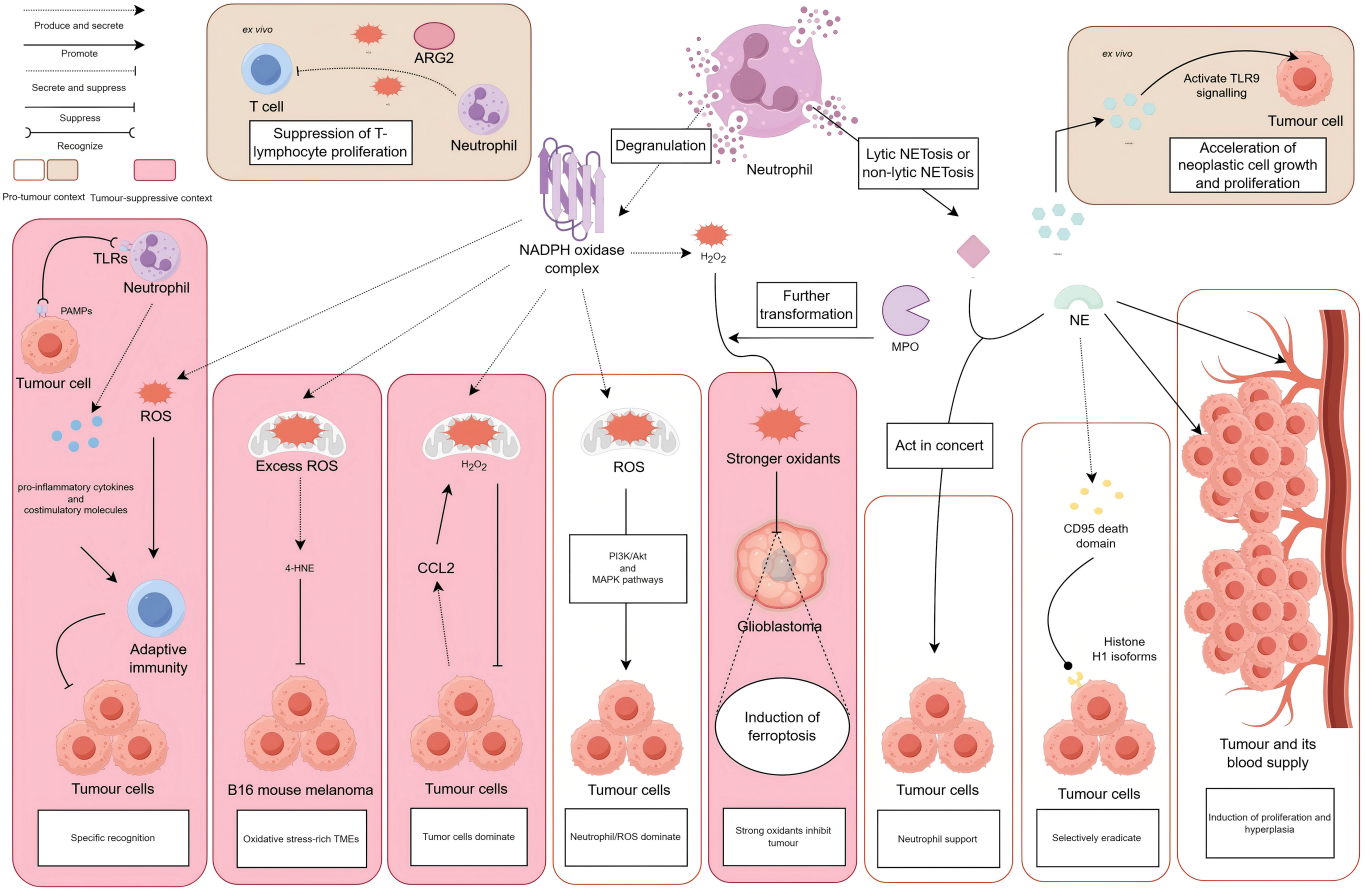
Neutrophils perform dual antitumoural and protumoural roles, either through degranulation that releases ROS, NO, and ARG2, or via NETosis that liberates NET components such as MPO and NE. *In vitro*, tumour-associated neutrophils produce ROS, NO, and ARG2, indicating a capacity for T-cell immunosuppression. Neutrophil-derived NETs stimulate HMGB1 release, which activates TLR9-dependent signalling in cancer cells and accelerates tumour proliferation. *In vivo*, TLR engagement on neutrophils by PAMPs triggers ROS production, proinflammatory cytokine secretion, and costimulatory molecule upregulation, together initiating adaptive antitumour immunity. Within oxidative stress-rich TMEs, excessive ROS from neutrophils initiates polyunsaturated fatty acid peroxidation cascades, yielding 4-HNE that suppresses B16-F10 melanoma growth. Tumour-secreted CCL2 mediates neutrophil H_2_O_2_ production to inhibit metastasis. In contrast, neutrophil-derived ROS activates PI3K/Akt and MAPK signalling pathways, thereby promoting tumourigenesis. MPO converts H_2_O_2_ into more potent oxidants that accumulate in glioblastoma cells alongside iron-mediated lipid peroxides, inducing ferroptosis and tumour necrosis. NE acts synergistically with MMP-9 to promote tumour migration and invasion. Proteolytic cleavage by NE releases CD95 death domains that bind histone H1 variants on malignant cells, selectively triggering apoptosis without harming normal tissues. Paradoxically, neutrophil-secreted NE also enhances tumour proliferation and angiogenesis.

As presented in **Figure 2**, neutrophils can exert dual regulatory roles in tumour immunity (14), depending on the microenvironmental context (35). Understanding the molecular basis of neutrophil-tumour interactions may support the development of targeted therapies. At present, TANs are classified using either the MDSC framework or the simplified N1-N2 paradigm (36). Both M-MDSCs and G/PMN-MDSCs represent immunosuppressive subsets (29). G/PMN-MDSCs often coexist with neutrophils in pathological settings and may also arise via transformation pathways from neutrophils (37). Human neutrophils are typically defined as CD14^−^CD11b^+^CD15^+^CD66b^+^ cells, distinguishing them from PMN-MDSCs on the basis of surface markers (35, 38, 39). Moreover, MDSCs can differentiate into granulocytes ex vivo, with granulocytes including neutrophils and other granular leucocytes (40).

Cytokine- and disease-driven stimuli polarise neutrophils into pro-tumourigenic or antitumourigenic states during tumourigenesis (41). Following the convention for macrophage polarisation (M1/M2) (42, 43), neutrophils can be broadly classified as N1 (anti-tumour) or N2 (pro-tumour). Although not all studies explicitly adopt this nomenclature, their findings are generally interpreted within this framework. N1 neutrophils are induced by IFN-β, interferon-gamma (IFN-γ), or granulocyte-macrophage colony-stimulating factor (GM-CSF) (38), with interferon-alpha (IFN-α) or IFN-β alone also sufficient to promote the phenotype (43, 44). By contrast, TGF-β alone induces N2 polarisation (40, 43). N2 neutrophils show upregulation of arginase 1 (ARG1), CCL17, and CXCL14, and downregulation of CXCL10, CXCL13, CCL6, tumour necrosis factor (TNF), intercellular adhesion molecule 1 (ICAM1), and endothelial-associated proteins (38, 43). Functionally, N2 neutrophils enhance chemotaxis, increase cytotoxicity, and suppress host immune responses. When cytokines such as TGF-β or IFNs are tested in combination with GM-CSF or G-CSF, tumour growth may either accelerate or show temporary control, reflecting the central role of cytokines in neutrophil recruitment (45–47). The precise origin of N2 neutrophils remains unresolved. It is unclear whether they derive from MDSCs recruited into tumours and subsequently transformed, or from circulating neutrophils that acquire the N2 phenotype under the influence of tumour-derived TGF-β (40, 43). Clarifying this issue may provide opportunities for therapeutic interventions targeting either systemic circulation or the tumour itself (48).

Neutrophils influence tumours in both directions using ROS/RNS and NETs (49, 50). ROS and RNS can function as signalling intermediates that sustain invasiveness in tumour cells, but can also induce senescence or apoptosis, serving as antitumour effectors (51). Accumulation of RNS contributes to oxidative stress, a hallmark of malignancy and a driver of tumour progression. NETs are fibrous DNA-histone structures decorated with granular proteins. They include proteases such as cathepsin G (CG), neutrophil elastase (NE), proteinase 3 (PR3), and MMP-2/9; enzymes such as MPO; and proteins including lactotransferrin, leucine leucine-37 (LL-37), calprotectin, bactericidal/permeability-increasing protein, pentraxin 3, citrullinated histone H3 (CitH3), interleukin-6 (IL-6), and Interleukin-17A (IL-17A) (46, 52–54). NETs and their components contribute to tumour progression via diverse mechanisms but in some contexts may also exert tumour-suppressive effects. Since neutrophils can produce similar effects via different molecules, or use the same mediator through different pathways, their functions in tumours remain highly context dependent. **Table 2** summarises the principal neutrophil-derived molecules and their dual roles in tumour regulation.

**Table 2:** Cytokines and factors with dual roles in tumour progression and suppression.

Cytokine/factor	Biological effect	Molecular mechanism
CD32a, CD16b	Kill the tumour cells	Cytotoxic effect (55)
TNF-α	Dose-dependent dichotomous effects on neutrophil function and tumour cell viability	Immune regulation, targeting tumour cells (39, 45, 56, 58)
CCL2	Suppresses tumour dissemination and exerts tumouricidal activity	ROS generation; MET pathway activation (58)
CCL2/CCL17	Modulates immune mechanisms to promote tumour cell proliferation, progression, and therapy resistance	Enhances migration of HCC cells and Tregs (40)
NETs	Dual role: • antitumour (39, 47, 65, 71) • Pro-tumour (72, 73, 76, 77)	Physical barrier formation (39); Immune system modulation (47, 51, 65)
MPO	Mediates tumour cell cytotoxicity (58)	Generates reactive ROS/RNS (58)
NE	Selectively eradicates malignant cells whereas providing pro-survival signals for TANs (75)	Cleaves CD95 death domain (61); Promotes angiogenesis (75)
NE ^+^ MMP-9	Angiogenesis promotion	Facilitates tumour cell migration/invasion (79)
MMP-2/MMP-9	Promotes tumour cell proliferation, invasion, and metastasis	ECM remodelling (39, 63)
Histones	Tumour suppression	Tumour antigen recognition; Immune regulation (60)
Integrins	Tumour-promoting	Dormant tumour cell reactivation (72)
PAD4	Promotes melanoma, CRC liver metastases, and pancreatic tumour growth (43, 73, 74)	Histone citrullination-induced NETosis (43, 82); Cancer-associated fibroblast(CAF) interaction (74)
TLRs	antitumour immunity	Adaptive immune activation (58)
BAFF/APRIL/IL-21	antitumour immunity	Enhances adaptive immune responses (66)
CCL3/CXCL9-10/IL-12/TNF-α/GM-CSF	antitumour immunity	Cytotoxic lymphocyte activation (39, 43, 71)
Bv8/MMP-9/VEGF-A	Tumour-promoting	Vascular niche establishment (75)
IL-6	Promote the survival of tumour cells	Anti-apoptotic signalling (88)
IL-8 ^+^ Neutrophils	Mediates tumour growth, angiogenesis, metastasis, extravasation, and endothelial anchoring (93)	MCAM/MUC18 interaction (melanoma) (93)
IL-8 ^+^ ICAM-1	Tumour-promoting	Individual effects: Maintains tumour cell dissemination, migration, and extravasation capacity (76, 81) Cooperative effects: Mediates coordinated tumour extravasation and endothelial anchoring (82)
SAFP	Maintain higher invasiveness and metastatic capacity of tumour cells (79)	EMT induction (78, 79); NET formation
LTB4	Metastatic niche formation	Selectively expand subgroups of cancer cells with high tumourigenic potential (76)
BMP2/TGF-β2	Increased expression of HCC tumour stem cells, promoting colonisation and tumour growth	Higher levels of CXCL chemokine 5 (CXCL5) were secreted, and more TAN infiltration increased the expression of HCC tumour stem cells, promoting colonisation and tumour growth (40)
Matrix metalloproteinases	Metastasis/immune evasion	ECM degradation (43)
HMGB1	Increases tumour aggressiveness	TLR9 pathway activation (72, 73, 77)
CXCR4	Acquired the ability to generate early angiogenesis, genotoxic damage, and viral infections	In areas of the lungs rich in CXCL12; special reactions occur in the bone marrow and spleen (97)
ARG2	Immune suppression	Inhibits T-cell proliferation (38, 95, 96)

#### 2.2.1 Antitumour effects

Early studies showed that neutrophils exert antitumour effects through multiple mechanisms, including direct tumouricidal activity and ADCC (41). These functions are mediated by cytokine secretion, release of effector molecules, or modulation of the TME. Collectively, they induce tumour cell apoptosis, inhibit migration, regulate adaptive immune responses, and enhance host antitumour immunity (1). TANs express Fc gamma receptor IIa (FcγRIIa, CD32a), enabling recognition and elimination of immunoglobulin G (IgG)-opsonised tumour cells through antigen-antibody interactions. Fc gamma receptor IIIb (CD16b) expression is also essential: blockade with anti-FcγRIIIb immunoglobulin G1 (IgG1) antibodies abolishes neutrophil-mediated cytotoxicity (55). Neutrophils can further induce tumour cell apoptosis by Fas receptor (FasR)-Fas ligand (FasL) engagement (56). However, some tumours evade this pathway by downregulating FasL, suggesting it may not represent the predominant neutrophil-mediated antitumour mechanism.

##### 2.2.1.1 Secretion of specialised substances or upregulation of endogenous molecules

In murine models, neutrophils secrete tumour necrosis factor-alpha (TNF-α), which exerts context-dependent effects. TNF-α can support tumour growth but also induces neutrophil apoptosis, implying transient antitumour potential (39, 45). In clinical settings, therapeutic TNF-α has shown limited efficacy. Given its tumour-promoting actions, TNF-α blockade, either alone or in combination with immune checkpoint blockade (ICB), has demonstrated antitumour promise (56). Inhibiting TNF-driven activation-induced T cell death, for example, improves ICB efficacy. In oxidative stress-rich TMEs, neutrophils generate excess ROS, triggering lipid peroxidation cascades in polyunsaturated fatty acids and producing 4-hydroxynonenal (4-HNE), which suppresses B16 mouse melanoma, Fluorescence-selected subline 10 (B16-F10) melanoma growth (57). Tumour-derived CCL2 also stimulates neutrophil production of hydrogen peroxide (H2O2), restricting metastatic spread. In addition, activation of the MET signalling pathway, mediated by the receptor tyrosine kinase MET and its ligand hepatocyte growth factor (HGF), enhances neutrophil NO release, which amplifies oxidative stress and promotes tumour cell killing (58). Simultaneously, elevated CCL2 from breast cancer cells recruits IFN-γ-producing monocytes, which then induce TMEM173 expression on neutrophils, enhancing their cytotoxicity (59).

##### 2.2.1.2 NETs under antitumour conditions

NETs can function as physical barriers that limit tumour dissemination. Their histone components exert direct cytotoxic effects on tumour cells (60). Among NET proteins, MPO generates ROS/RNS that induce apoptosis. In glioblastoma, MPO amplifies iron-dependent lipid peroxidation, triggering ferroptosis and necrosis (58). NE also hydrolytically releases the CD95 death domain, which interacts with histone H1 subtypes on cancer cell surfaces, selectively killing malignant cells whereas sparing normal tissues (61). Evidence suggests that NET components may exert either antitumour or pro-tumour effects depending on their molecular interactions and context (39, 51–53, 58, 60–63).

##### 2.2.1.3 Immunomodulatory functions: cytotoxic effects and release of bioactive substances

Pattern recognition receptors (PRRs), particularly Toll-like receptors (TLRs), enable neutrophils to detect pathogen-associated molecular patterns (PAMPs). Engagement of these receptors induces ROS/RNS production, release of pro-inflammatory cytokines, and upregulation of costimulatory molecules, collectively priming adaptive immunity (57). Neutrophils further modulate adaptive responses through NET formation, which serves as an immunostimulatory platform that enhances lymphocyte activation (64). Splenic neutrophils secrete B cell-activating factor (BAFF), a proliferation-inducing ligand (APRIL), and IL-21, activating marginal zone (MZ) B cells and promoting class switching, somatic hypermutation, and antibody production (65). As APCs, neutrophils express MHC class II (MHCII) and can prime CD4^+^ T cells both *in vitro* and *in vivo* (66). They also secrete chemoattractants (e.g., CCL3, CXCL9, CXCL10) and cytokines (e.g., interleukin-12 [IL-12], TNF-α, GM-CSF, VEGF) that recruit and activate CD8^+^ T cells, thereby enhancing cytotoxicity. Through cell-cell contact and TNF-α release, neutrophils activate dendritic cells (DCs), enhancing antigen presentation and T cell priming (43). In addition, neutrophils stimulate macrophages, NK cells, and T cell subsets, amplifying antitumour immunity (39, 66). NETs further enhance T cell activation by lowering the antigen threshold required for priming, thereby boosting adaptive immune responses (47). Collectively, these findings establish neutrophils not only as innate effector cells but also as potent modulators of adaptive antitumour immunity.

##### 2.2.1.4 Clinical applications as delivery vectors

This represents a clinically distinct approach from conventional neutrophil-mediated antitumour mechanisms, exemplified by their synergistic use with oncolytic viruses (OVs) (44). OVs selectively infect and lyse tumour cells whereas stimulating host immunity. They include DNA viruses (e.g., adenovirus [Adv], herpes simplex virus [HSV]) and RNA viruses (e.g., measles virus [MV]) (44). Their antitumour activity may occur naturally or be enhanced through genetic engineering. OVs replicate selectively within tumours, deliver therapeutic genes, and remodel the immunosuppressive TME through multiple mechanisms (67).

For example, in clinical studies combining neutrophils with vaccinia virus (VACV), intravenous administration of recombinant VACV engineered to express interleukins (ILs) enhanced neutrophil infiltration and migration. Although VACV directly targeted tumour cells, neutrophils contributed additional antitumour activity, collectively suppressing malignant mesothelioma growth modified vaccinia ankara (MVA) (68). With vesicular stomatitis virus (VSV), pretreatment using the neutrophil-depleting rat anti-mouse lymphocyte antigen 6 complex, locus G/lymphocyte antigen 6 complex, locus C (Ly-6G/Ly-6C) monoclonal antibody clone RB6-8C5 (RB6-8C5) antibody (44) both recruited neutrophils and compensated for replication suppression inherent to VSV, thereby optimising the TME (69). In parallel, VSV combined with neutrophils induced disruption of tumour vasculature, attributable to VSV’s natural tropism for tumour vessels and neutrophil-mediated fibrin deposition and clot initiation (70). A key limitation of this strategy, however, is that excessive neutrophil recruitment suppresses VSV replication and dissemination, diminishing therapeutic efficacy (44).

#### 2.2.2 Pro-tumour effects

##### 2.2.2.1 Promotion of cancer cell activation, progression, and dissemination

NETs, induced by pro-inflammatory stimuli, remodel laminin proteolytically, activating integrins that stimulate dormant cancer cell proliferation and accelerate tumour progression (43, 71, 72). As summarised in **Table 2**, neutrophils promote melanoma, colorectal cancer (CRC) liver metastasis, and pancreatic cancer progression via peptidylarginine deiminase 4 (PAD4) and citrullinated histone synthesis and secretion (43, 73, 74). During tumour development, neutrophils support malignant cells by promoting angiogenesis at primary and metastatic sites, thereby supplying nutrients, increasing metabolic burden, and complicating therapy. VEGF secretion is central to this process. vascular endothelial growth factor A (VEGF-A) recruits pro-angiogenic CD11b^+^Gr-1^+^ (granulocyte receptor-1-positive) CXCR4^+^ neutrophils, enhancing post-transplant islet revascularisation and functional integration (75). Neutrophils also reinforce VEGF activity by releasing proteins such as prokineticin 2 (PROK2, also known as Bv8) and MMP-9 (75). NE has likewise been shown to drive tumour proliferation and angiogenesis (75). MDSCs, a heterogeneous immunosuppressive population, expand in the spleen and peripheral blood of cancer patients. Existing as monocytic (lymphocyte antigen 6 complex, locus C positive [Ly6C^+^]) or granulocytic (Lymphocyte Antigen 6 Complex, Locus G Positive [Ly6G^+^]) subsets, MDSCs exert systemic immunosuppressive and pro-angiogenic functions (43). TANs are closely related to MDSCs (39, 43), suggesting that modulation of splenic and circulating MDSCs may provide therapeutic benefit.

Cancer cell dissemination describes the spread of tumour cells or their metabolites within tissues, enabling expansion at the primary site. Dissemination occurs through passive diffusion (concentration gradients) or active diffusion (environmental changes). M2 macrophages and activated neutrophils secrete interleukin-8 (IL-8), sustaining tumour dissemination and migration (76). Increased motility and detachment from the primary lesion facilitate more extensive spread.

#### 2.2.2.2 Promotion of tumour cell activation, detachment, and circulatory entry

Metastatic tumour cells demonstrate enhanced migration, with neutrophils playing a central role in this process (77). Neutrophils promote EMT, which confers invasive and migratory potential (78). For instance, hypopharyngeal tumour cells undergo partial EMT upon stimulation by PMNs, with more complete transformation following Staphylococcus aureus exposure, leading to greater invasiveness. In head and neck squamous cell carcinoma (HNSCC), interactions among bacteria, neutrophils, and tumour cells accelerate the emergence of mesenchymal, metastasis-prone phenotypes (79). After EMT, tumour cells undergo morphological, molecular, and functional changes, transitioning from localised lesions to invasive malignant forms (78). They may further enhance migration by expressing surface chemoattractants or clustering with neutrophils (tumour cell-PMN complexes, TC-PMNs) via vascular cell adhesion molecule-1 (VCAM-1) interactions (80). Neutralisation of IL-8 reduces extravasation of PMN-associated tumour cells, whereas ICAM-1 overexpression provides an alternative pathway for melanoma extravasation (76, 81).

Circulating tumour cells (CTCs) undergoing EMT display heightened metastatic potential. Most CTCs exhibit incomplete EMT, co-expressing epithelial and mesenchymal markers yet showing increased malignancy (45). In BC, differential gene expression between CTCs and their associated neutrophils supports cell cycle progression, accelerating metastatic seeding (82). Neutrophils also facilitate pulmonary invasion by CTCs, enhancing survival, proliferation, invasion, and extravasation through secretion of cytokines and chemoattractants as well as NET formation. They additionally condition the pre-metastatic niche and remodel the TME to support colonisation (76, 82, 83).

#### 2.2.2.3 Maintenance or enhancement of tumour invasion, metastasis, and colonisation

Tumour invasion refers to the penetration of the basement membrane by tumour cells at the primary site and infiltration into adjacent tissues. Metastasis denotes dissemination to distant organs, where colonisation refers to the proliferation of tumour cells following “settlement” at secondary sites. *Ex vivo* studies in MC38 colon cancer cell lines and hepatic metastasis models demonstrated that NETs promote release of high-mobility group box 1 (HMGB1), which activates Toll-like receptor 9 (TLR9) signalling in cancer cells. This enhances proliferation, adhesion, migration, and invasion, thereby increasing tumourigenic potential (72, 73, 77). *In vivo*, elevated NET levels correlate with poor prognosis in breast, colorectal, gastric, lung, and pancreatic cancers, and are strongly associated with liver, lung, and omental metastases (46). Once formed, NETs use their fibrous networks to capture CTCs, facilitating metastatic spread (76). Clinical samples from triple-negative breast cancer patients show that NETs can drive tumour metastasis. Experiments with NK cells indicate that NETs’ physical barrier contributes to their pro-tumour effects (84). NETs create a favorable microenvironment for ovarian cancer growth and metastasis, aiding its colonization in the omentum and the hepatic colonization of colorectal, lung, and breast cancers (85). At the molecular level, neutrophil elastase and matrix metalloproteinase-9 in NETs can trigger dormant lung cancer cells to proliferate through laminin remodelling (86). NE and MMP-9 cooperate to promote tumour migration and invasion (79). Similarly, MMP-2 and MMP-9 remodel the ECM, further supporting tumour cell proliferation and metastasis (38, 39). Neutrophil-secreted IL-6 enhances tumour cell survival by conferring resistance to apoptosis (87, 88). Furthermore, β2 integrins (CD18) on IL-8^+^ neutrophils interact with ICAM-1 on melanoma cells, anchoring tumour cells to vascular endothelium and facilitating extravasation (82). Leukotriene B4 (LTB4) has also been shown to enhance colonisation by expanding highly tumourigenic cancer cell subsets (76).

Other mechanisms include neutrophil secretion of cytokines such as CCL2 and CCL17, which modulate immune responses to promote tumour growth, progression, and drug resistance (40). Sorafenib is a treatment for hepatocellular carcinoma (HCC) that enhances its anticancer effects by inhibiting CCL2 and CCL17 transcriptional regulators in neutrophils (89). In HCC, neutrophils activate stem cell activity through secretion of bone morphogenetic protein 2 (BMP2) and transforming growth factor-beta 2 (TGF-β2) and downstream mediators (40). Neutrophil-derived CXCR4-dependent transformation has also been implicated in tumour promotion within the lungs, bone marrow, and spleen (90). Additionally, neutrophils support tumour metastasis by producing matrix-degrading enzymes while simultaneously suppressing anti-tumour immune responses (43). ROS serve as signalling molecules that activate phosphoinositide 3-kinase/protein kinase B (Phosphatidylinositol 3-Kinase [PI3K]-AKT Serine/Threonine Kinase [AKT]) and mitogen-activated protein kinase (MAPK) pathways, driving tumour progression (91, 92). Furthermore, interactions between melanoma cell adhesion molecule (melanoma cell adhesion molecule [MCAM, also termed MUC18 or CD146]) on tumour surfaces and neutrophil-derived IL-8 promote melanoma proliferation, angiogenesis, and metastasis (93).

#### 2.2.2.4 Neutrophils and their synthesised/secreted substances suppress tumour-specific and non-specific immune functions

Experimental evidence indicates that targeting neutrophil activity may improve therapeutic efficacy in cancer. In non-small cell lung cancer (NSCLC), modulation of neutrophil function has been shown to enhance responses to immune checkpoint inhibitors (ICIs) (94). Transcriptomic analyses of neutrophils from the spleen and blood of breast cancer-bearing mice revealed that tumour-induced neutrophils produce ROS, NO, and arginase 2(ARG2), suppressing T-cell proliferation *ex vivo* and demonstrating an immunosuppressive N2-like phenotype (38, 95, 96). In renal cell carcinoma and NSLC patients, high ARG production in neutrophils can suppress T cell functions, akin to the effect of M2 tumour-associated macrophages (TAMs) (43, 59). TAMs, an immunosuppressive macrophage subset within the TME (43). Moreover, stimulation of tumour cells with Staphylococcus aureus filtrate preparation (SAFP) indirectly activates PMNs, inducing NET formation. These NETs form a barrier that impedes NK cell surveillance of partial epithelial-mesenchymal transition (p-EMT) tumour cells, enabling immune evasion and sustaining their invasive and metastatic potential (79).

## 3 HNA system and tumour

**Figure 3:**
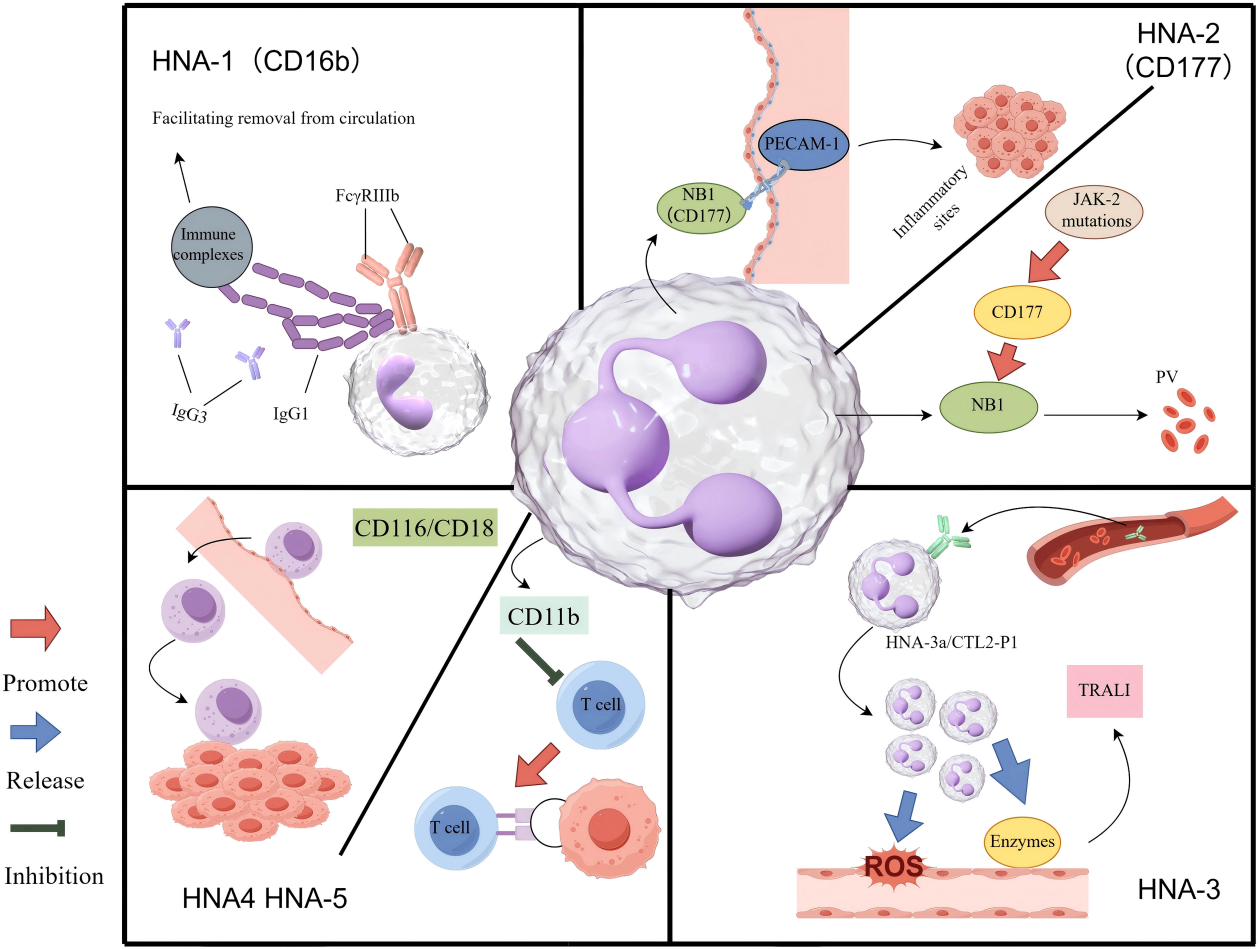
The mechanism of HNA antigen system in inflammation and tumour. HNA-1: The FcγRIIIb receptor engages the Fc region of polymeric immune complexes through its membrane-proximal domain. In resting neutrophils, FcγRIIIb is predominantly utilized to detect immune complexes and facilitate their clearance from the circulatory system. HNA-2: Upon activation, neutrophils express HNA-2 (also referred to as NB1 glycoprotein) on their surface, which interacts with PECAM-1 on vascular endothelial cells in a coordinated manner. This interaction facilitates firm adhesion to the vessel wall, followed by cytoskeletal reorganization and transendothelial migration into tissues, where neutrophils play a role in resolving inflammation or infection. In individuals with a JAK2 mutation, hyperactivation of the CD177 gene, which regulates HNA-2 expression, leads to excessive production of NB1 glycoprotein (HNA-2). This overexpression may alter neutrophil migratory behaviour, potentially contributing to the pathogenesis of polycythemia vera. HNA-3: Circulating anti-HNA-3a antibodies interact with neutrophils expressing the HNA-3a/CTL2-P1 antigen. This antigen-antibody interaction induces neutrophil agglutination and activates NADPH oxidase. Consequently, the activated neutrophils release ROS and proteolytic enzymes, resulting in endothelial damage, compromised vascular integrity, and edema, ultimately leading to TRALI. HNA-4 & HNA-5: The CD11b/CD18 integrin facilitates the adhesion of immune cells to the vascular endothelium and supports their migration into tumour sites. On TANs, the expression of CD11b may contribute to immune evasion, in part by suppressing T-cell function.

The HNA system holds considerable clinical relevance in neutrophil biology. As summarised in **Table 3**. Non-neutrophil-specific groups, including HNA-3, HNA-4, and HNA-5, are predominantly associated with transfusion-related complications, neutropenia, HSCs transplant rejection, and renal allograft rejection (97). In addition, certain antigen subtypes regulate neutrophil function and influence the tumour immune microenvironment (TIME), thereby affecting tumour initiation, progression, treatment response, and prognosis (**Figure 3**).

**Table 3:** Biochemical characteristics, genetic expression and clinical significance of HNA antigen system.

HNA antigen system	Allele	Nucleotide position of corresponding Allele	Amino acid position in glycoprotein	Epitope	Glycoprotein	Function
		Conventional positions (141) (147) (227) (266) (277) (349)			FccRIIIb,CD16	
HNA-1	FCGR3B*01 FCGR3B*02 FCGR3B*03 FCGR3B*04 FCGR3B*05 FCGR3B*null	108G 114C 194A 233C 244G 316G 108C 114T 194G 233C 244A 316A 108C 114T 194G 233A 244A 316A 108G 114C 194A 233C 244G **316A** 108C 114T 194G 233C **244G** 316A No alleles	36Arg 38Leu 65Asn 78Ala 82Asp 106Val 36Ser 38Leu 65Ser 78Ala 82Asn 106Ile 36Ser 38Leu 65Ser 78Asp 82Asn 106Ile 36Arg 38Leu 65Asn 78Ala 82Asp **106Ile** 36Ser 38Leu 65Ser 78Ala **82Asp** 106Ile No glycoprotein (gp)	HNA-1a HNA-1b HNA-1d‡ HNA-1b HNA-1c HNA-1a HNA-1b|| HNA-1 null	No gp	Low-affinity IgG receptor involved in immune complex clearance and phagocytosis (98).
HNA-2	CD177	Allelic variation of this gene does not code for different serological phenotypes Differential mRNA splicing: HNA-2 negative phenotype		HNA-2 HNA-2null	CD177 No gp	Plays a role in immunoregulation and inflammatory responses (99), contributes to neutrophil function and myeloid cell proliferation. Promotes neutrophil transendothelial migration (99); co-expressed with PR3 (99).
HNA-3	SLC44A2*01 SLC44A2*02 SLC44A2*03	451C 455G 451C 455A 451T 455G	151Leu 152Arg 151Leu 152Gln 151Phe 152Arg	HNA-3a HNA-3b HNA-3a||		
HNA-4	ITGAM*01 ITGAM*02	230G 230A	Arg61 His61	HNA-4a HNA-4b	CD11b	Plays a role in leucocyte adhesion and phagocytosis (100).
HNA-5	ITGAL*01 ITGAL*02	2372G 2372C	Arg766 Thr766	HNA-5a	CD11a	Leucocyte adhesion molecule (98)

‡HNA-1d is the antithetical epitope of HNA-1c.

||Variations in reactivity with human antisera may be observed.

A single allele may encode multiple epitopes (e.g., FCGR3B03 encodes both HNA-1b and HNA-1c; FCGR3B02 encodes HNA-1b and HNA-1d). Conversely, an antigen may be encoded by multiple alleles (e.g., HNA-1a is encoded by both FCGR3B01 and FCGR3B04) (4).

In this table of HNA alleles and antigens, the underlined characters/bolded (such as "106Ile" of FCGR3B03, "316A" of FCGR3B04, "244G" and "82Asp" of FCGR3B05, "HNA-3a§" of SLC44A203) represent the nucleotide/amino acid changes at these positions as the "key variant sites" of the allele, which are the core differences distinguishing this allele from other subtypes. Specifically:For example, "106Ile" of FCGR3B03: This indicates that the amino acid at position 106 of this allele is isoleucine (Ile), which is the differentiating site from other FCGR3B subtypes (such as 01/*02);"316A" of FCGR3B*04: This represents that the nucleotide at position 316 of this allele is A (adenine), a unique nucleotide variation of this allele;Similarly, the underlined annotations such as "82Asp" and "244G" are all characteristic nucleotide/amino acid sites that distinguish this allele from other subtypes, serving as the core identifiers of this allele.

### 3.1 HNA-1

#### 3.1.1 Biochemical characteristics, genetic expression, and clinical significance

The carrier protein of HNA-1 is Fcγ receptor IIIb (FcγRIIIb, CD16b), which is anchored to the cell membrane via glycosylphosphatidylinositol (GPI) (101). FcγRIIIb can be shed from the neutrophil surface upon stimulation by chemoattractants such as formyl-methionyl-leucyl-phenylalanine (fMLP) or G-CSF. Currently identified HNA-1 subtypes include HNA-1a, HNA-1b, HNA-1c, and HNA-1d, encoded by allelic variants of the Fc fragment of IgG receptor IIIb (FCGR3B) gene that exhibit extensive polymorphism. HNA-1a and HNA-1b differ by five single-nucleotide polymorphisms (SNPs), resulting in four amino acid substitutions, whereas HNA-1c arises from a single-nucleotide variant (266C>A) (98). HNA-1d is defined by the Alanine-78 through Asparagine-82 (Ala78-Asn82) sequence encoded by FCGR3B02, whereas FCGR3B03 carries an Ala78Asp (alanine-to-aspartate substitution at position 78) mutation that abolishes HNA-1d expression, sharing allelic identity with HNA-1b (102).

Neutrophil development is a continuous process regulated by transcription factors and epigenetic mechanisms, identifiable by distinct progenitor states and lineages (103). In human bone marrow, neutrophil progenitors like NCP5 and NCP6, which precede promyelocytes, have unique gene expressions and differentiation potential (104). A key study found that human metamyelocytes and band cells naturally have immunosuppressive properties (105), highlighting the link between developmental stages and immune functions. FcγRIIIb (CD16b) expression starts at the myelocyte stage, leading to high HNA-1 alloantigen levels in mature neutrophils, while remaining low in early progenitors like NCP5 and NCP6 (98). In paroxysmal nocturnal haemoglobinuria (PNH), the absence of glycosylphosphatidylinositol (GPI)-anchored proteins leads to deficient FcγRIIIb and HNA-1 expression on granulocytes (106, 107). The distribution of HNA-1 alleles varies markedly among populations. HNA-1a is highly prevalent in East Asians, whereas HNA-1b and HNA-1d are more common in Europeans. HNA-1c is rare in European and African populations and virtually absent in East Asians, including Chinese, Japanese, and Korean cohorts (108) Clinically, HNA-1 is implicated in neonatal alloimmune neutropenia (NAIN), autoimmune neutropenia (AIN), and transfusion-related acute lung injury (TRALI).

#### 3.1.2 Possible mechanisms affecting the TME

HNA-1 (CD16b) likely contributes to tumour immunity through roles in immune complex clearance and phagocytosis. Certain tumours, including acute myeloid leukaemia (AML) and solid tumours, may evade immune surveillance by modulating Fc gamma receptor (FcγR) expression (109). FcγRIIIb functions as a low-affinity receptor for IgG1 and immunoglobulin G3 (IgG3), binding polymeric immune complexes but not monomeric IgG. Resting neutrophils engage FcγRIIIb to bind immune complexes, facilitating their removal from circulation (98). Polymorphisms in FCGR3B influence receptor affinity: neutrophils homozygous for HNA-1a exhibit higher affinity for IgG3 and enhanced phagocytic capacity, whereas HNA-1b carriers demonstrate weaker activity (110). These differences may modulate neutrophil-mediated cytotoxicity against tumour cells.

In AML, HNA-1 antibodies are strongly associated with transfusion-related risks, particularly TRALI, which remains a major complication in patients undergoing chemotherapy or HSCs transplantation. This risk is heightened by exposure to blood products containing HNA-1 antibodies, often derived from multiparous female donors (111). Whether HNA-1 is expressed directly on AML cells remains unresolved.

### 3.2 HNA-2

#### 3.2.1 Biochemical characteristics, genetic expression, and clinical significance

HNA-2, also known as the NB system, is a neutrophil-specific antigen carried by the NB1 glycoprotein, encoded by the CD177 gene. CD177 encodes a 58–64 kDa GPI-anchored glycoprotein belonging to the Ly-6/urokinase plasminogen activator receptor (uPAR)/snake toxin family, containing three N-linked glycosylation sites and two cysteine-rich domains. The gene is located on chromosome 19q13.31, spans ~9.5 kb, and comprises nine exons encoding 437 amino acids.

Unlike most HNA-2-positive individuals, who express this glycoprotein only on a variable fraction of granulocytes, HNA-2-negative individuals (equivalent to the HNA-2 null phenotype) completely lack its expression (98). The proportion of HNA-2-positive neutrophils varies markedly among individuals, and dysregulated expression has been implicated in diseases such as myelodysplastic syndromes (MDS), chronic myeloid leukaemia (CML), and gastric cancer(GC) (112). A unique feature of HNA-2 is that its expression begins at the myelocyte stage and remains restricted to neutrophil subpopulations, including myelocytes, metamyelocytes, and mature segmented neutrophils (97).

HNA-2 is expressed in 97% of Caucasians, 95% of African Americans, and 89% of Japanese individuals, but only in neutrophil subpopulations, averaging 50-60% (113). Among blood cells, HNA-2 is uniquely expressed on neutrophils, localised to the plasma membrane, secondary granules, and secretory vesicles (114). Its expression is influenced by multiple factors, including sex (slightly higher in females), pregnancy, G-CSF stimulation, and severe bacterial infections. HNA-2 deficiency, observed in 3-5% of the population, arises mainly from abnormal CD177 mRNA splicing or transcriptional silencing and is strongly associated with myeloproliferative disorders (MPDs) (112, 115).

The clinical relevance of HNA-2 (CD177) spans transfusion medicine (TRALI, neonatal immune neutropenia), autoimmune disease [anti-neutrophil cytoplasmic antibody (ANCA) vasculitis], haematological malignancies [diagnosis and prognosis in myeloproliferative neoplasms (MPNs)], and the solid TME (potential carcinogenic functions). HNA-2, a key neutrophil antigen, is crucial for immune regulation, inflammation, neutrophil function, and myeloid cell growth (99, 112). In tumours, neutrophils have a dual role (116, 117): they can either inhibit tumour growth by activating T cells and inducing apoptosis or promote tumour progression by causing inflammation, enhancing angiogenesis, and suppressing immune responses (118). This dual function, divided into anti-tumour N1 and pro-tumour N2 phenotypes, highlights the adaptability of neutrophils in cancer immune regulation, influenced by microenvironmental signals (38).

#### 3.2.2 Mechanisms in haematological neoplasms

Polycythaemia vera (PV) is a chronic MPN characterised by clonal expansion of erythroid, megakaryocytic, and granulocytic lineages. HNA-2 is the only antigen markedly upregulated in a neutrophil subpopulation following G-CSF stimulation in PV or during bacterial infections (119).

The NB1 glycoprotein encoded by CD177 regulates neutrophil-endothelial adhesion via binding to platelet endothelial cell adhesion molecule-1 (PECAM-1, CD31), which is essential for transendothelial migration during inflammation (99). In PV, CD177 RNA expression is significantly increased, likely reflecting the Janus kinase 2 (JAK2) V617F mutation, a central driver of disease (5, 113).

Moreover, membrane-bound proteinase 3 (mPR3), a serine protease, is co-expressed with CD177 on a neutrophil subset. HNA-2 is required for PR3 anchoring, linking it to autoimmune pathology (120). Whereas HNA-2 is a primary alloantibody target in neutropenia-related conditions, mPR3 serves as a major autoantigen in ANCA-associated vasculitis (99, 119, 120). Elevated CD177 expression is observed in PV, essential thrombocythaemia, and primary myelofibrosis, as well as in secondary polycythaemia, CML, and MDS (115). Structurally, NB1 resembles polycythaemia rubra vera-1 (PRV-1) and uPAR, both overexpressed in PV granulocytes, suggesting a shared role in haematopoiesis (121).

#### 3.2.3 Potential association with gastric carcinogenesis

HNA-2 expression is significantly upregulated in aggressive GC tissues compared with normal gastric mucosa, correlating with tumour size, lymph node metastasis, and clinical stage (122). Immunohistochemistry reveals CD177 expression not only in tumour-infiltrating neutrophils but also in gastric adenocarcinoma cells, regardless of differentiation status (122). Given CD177’s structural similarity to uPAR, a mediator of cell adhesion and migration (123), it may regulate tumour adhesion and invasion in GC. HNA-2 overexpression may also polarise neutrophils towards pro-tumour phenotypes, promoting angiogenesis and immunosuppression within the TME. Although CD177 overexpression is linked to MPDs (e.g., PV) (115), its role in GC proliferation remains unconfirmed. Paradoxically, some data associate CD177 expression with improved prognosis in gastric adenocarcinoma (122), suggesting a dual role as both a carcinogenic driver and a prognostic biomarker (120, 122). Although its precise contribution to GC proliferation remains unconfirmed, the association of CD177 with inflammation, immune regulation (99), and cell migration supports its potential role in gastric tumour progression. Large-scale clinical studies are required to validate its prognostic and therapeutic value.

### 3.3 HNA-3

#### 3.3.1 Biochemical characteristics, genetic expression, and clinical significance

The HNA-3 antigen system comprises HNA-3a (formerly 5b) and HNA-3b (formerly 5a) and is not neutrophil-specific. HNA-3 antibodies can bind not only to neutrophils but also to lymphocytes, platelets, and other tissues, including inner ear structures (124). The target glycoprotein is choline transporter-like protein 2 (CTL2), encoded by Solute Carrier Family 44 Member 2 (SLC44A2). CTL2 contains five extracellular loops, with the HNA-3 antigen located on the first loop. A conformation-sensitive epitope at position 152 differentiates arginine (arginine at position 152 [Arg152], HNA-3a) from glutamine (glutamine at position 152 [Gln152], HNA-3b), defining the two polymorphisms (125). HNA-3a is encoded by the G allele, whereas HNA-3b is encoded by the A allele (126). Three genotypes/phenotypes exist: HNA-3aa homozygotes (55-64% of Caucasians), HNA-3ab heterozygotes (30-40%), and HNA-3bb homozygotes (≈5%) (127). Flow cytometry and polyclonal anti-CTL2 antibody assays indicate that HNA-3 genotypes influence antigen density on neutrophils but not overall CTL2 expression (128).

Unlike HNA-1 and HNA-2, which are neutrophil-restricted, HNA-3 exhibits multi-tissue expression, broadening its pathological relevance (120). The HNA-3a-negative phenotype occurs more frequently in East Asians (6-19%) than in Europeans (4-5%) and is rare in Africans (97). HNA-3a antibodies represent the leading cause of severe or fatal TRALI, due to combined effects on neutrophils and endothelial cells. Antibody type influences susceptibility: HNA-3ab heterozygotes are less prone to anti-HNA-3a-mediated TRALI than HNA-3aa homozygotes (128). In contrast, HNA-3b displays lower immunogenicity, and anti-HNA-3b alloantibodies are rarely clinically severe.

#### 3.3.2 Potential association with AML

AML patients are at high risk for TRALI because of frequent transfusion requirements. Anti-HNA-3a alloantibodies can cause fatal TRALI via complement activation, leading to pulmonary microvascular endothelial damage and non-cardiogenic pulmonary oedema. Transfusion of HNA-3a antibody-containing blood products, particularly platelets, can trigger acute respiratory distress syndrome (ARDS) in AML patients (129).

For example, a 66-year-old AML patient developed ARDS after platelet transfusion; post-mortem analysis revealed diffuse pulmonary oedema with leukaemic infiltration. Donor serum contained anti-HNA-3a antibodies, whereas the patient was HNA-3aa homozygous. Although findings suggested pulmonary leukaemic infiltration rather than typical TRALI, a role for HNA-3 antibodies could not be excluded (130). In another report, a paediatric AML patient developed fatal TRALI post-transplant after receiving platelets containing anti-HNA-3 antibodies, highlighting the potential for HNA-3 incompatibility to exacerbate pulmonary injury (111). AML-associated neutropenia, immune dysfunction, and chemotherapy may heighten vulnerability to HNA-3-mediated reactions. Following haematopoietic stem cell transplantation (HSCT), donor-derived granulocytes may interact with residual recipient antibodies (e.g., anti-HNA-3), worsening transplant-associated lung injury through complement activation (129).

### 3.4 HNA-4

#### 3.4.1 Biochemical characteristics, genetic expression, and clinical significance

HNA-4 is encoded by the integrin subunit alpha M (ITGAM) gene, with the antigenic determinant located on CD11b (αM subunit of the CD11b/CD18 integrin). The ITGAM gene resides on chromosome 16p11.2, spans 73 kb, and contains 30 exons encoding a 1,152-amino acid protein (97). The HNA-4a/HNA-4b polymorphism arises from an Arg61His substitution in CD11b, part of the CD11b/CD18 integrin (also known as Mac-1, CR3, or αMβ2 integrin). This integrin is expressed on neutrophils, monocytes, and NK cells, where it regulates leucocyte adhesion to endothelial cells and platelets, migration to inflammatory sites, phagocytosis, and oxidative burst activity (98, 131). Anti-HNA-4a antibodies can impair neutrophil adhesion and cause neonatal immune neutropenia. Conversely, the HNA-4b allele has been associated with increased risk of SLE (132).

#### 3.4.2 Role in the TME

The HNA-4 antigen is located on CD11b, a key integrin subunit with dual roles in tumour immunity. CD11b activation promotes antitumour responses and suppresses tumour growth (133). In resting leukocytes, including neutrophils, monocytes, and NK cells, CD11b/CD18 remains inactive but is rapidly upregulated upon activation. This facilitates immune cell adhesion to vascular endothelium, migration to tumour sites, phagocytosis, and oxidative burst (100). Beyond these effector functions, CD11b contributes to adaptive immunity. CD11b^+^ DCs and macrophages present tumour antigens to CD8^+^ T cells, thereby enhancing cytotoxic responses (132). Clinically, high CD11b expression on tumour-infiltrating myeloid cells correlates with prolonged patient survival. Conversely, CD11b also plays a central role in establishing an immunosuppressive TME. CD11b^+^ Gr-1^+^ MDSCs inhibit T-cell activity through secretion of ​​interleukin-10 (IL-10) and TGF-β, facilitating tumour immune evasion. Similarly, CD11b^+^ TAMs release VEGF and MMP-9, promoting angiogenesis and metastasis (134). During chronic inflammation, CD11b^+^ myeloid cells generate (ROS and proinflammatory mediators, which induce DNA damage and precancerous transformation, further linking inflammation to carcinogenesis.

### 3.5 HNA-5

#### 3.5.1 Biochemical characteristics, genetic expression, and clinical significance

HNA-5 is encoded by the integrin subunit alpha L (ITGAL) gene and located on the αL subunit (CD11a) of the leucocyte β2-integrin family (CD11a/CD18, also termed lymphocyte function-associated antigen 1 [LFA-1]) (125). Amino acid substitutions give rise to two subtypes: HNA-5a and HNA-5b. The ITGAL gene is positioned at 16p11.2 on chromosome 16, spans ~50.5 kb, and consists of 31 exons encoding a 1,170-amino acid protein (97). The CD11a/CD18 complex is expressed on all leucocytes and mediates leucocyte adhesion (98). The HNA-5a antigen is highly prevalent in European and East Asian populations but relatively uncommon in African populations (120). Although anti-HNA-5a alloantibodies have not been implicated in neutropenia, they have been detected in patients with aplastic anaemia. Interestingly, the HNA-5b polymorphism has been associated with enhanced immune responses to hepatitis B vaccination.

#### 3.5.2 Molecular basis and tumour-associated pathways

The HNA-5 antigen arises from polymorphisms in CD11a/CD18. This integrin complex plays important roles in leucocyte adhesion, migration, and infiltration, particularly of neutrophils and MDSCs. It contributes to tumour cell extravasation and metastasis by regulating ECM interactions. CD11b, another β2-integrin subunit, is highly expressed on TANs and MDSCs, where it promotes immune evasion by suppressing T-cell function, in part through PD-L1/2 signalling (134). By analogy, polymorphisms in HNA-5 may influence neutrophil functional polarisation and thereby shape tumour-associated immune responses.

## 4 Specific tumour type-based perspectives

**Figure 4:**
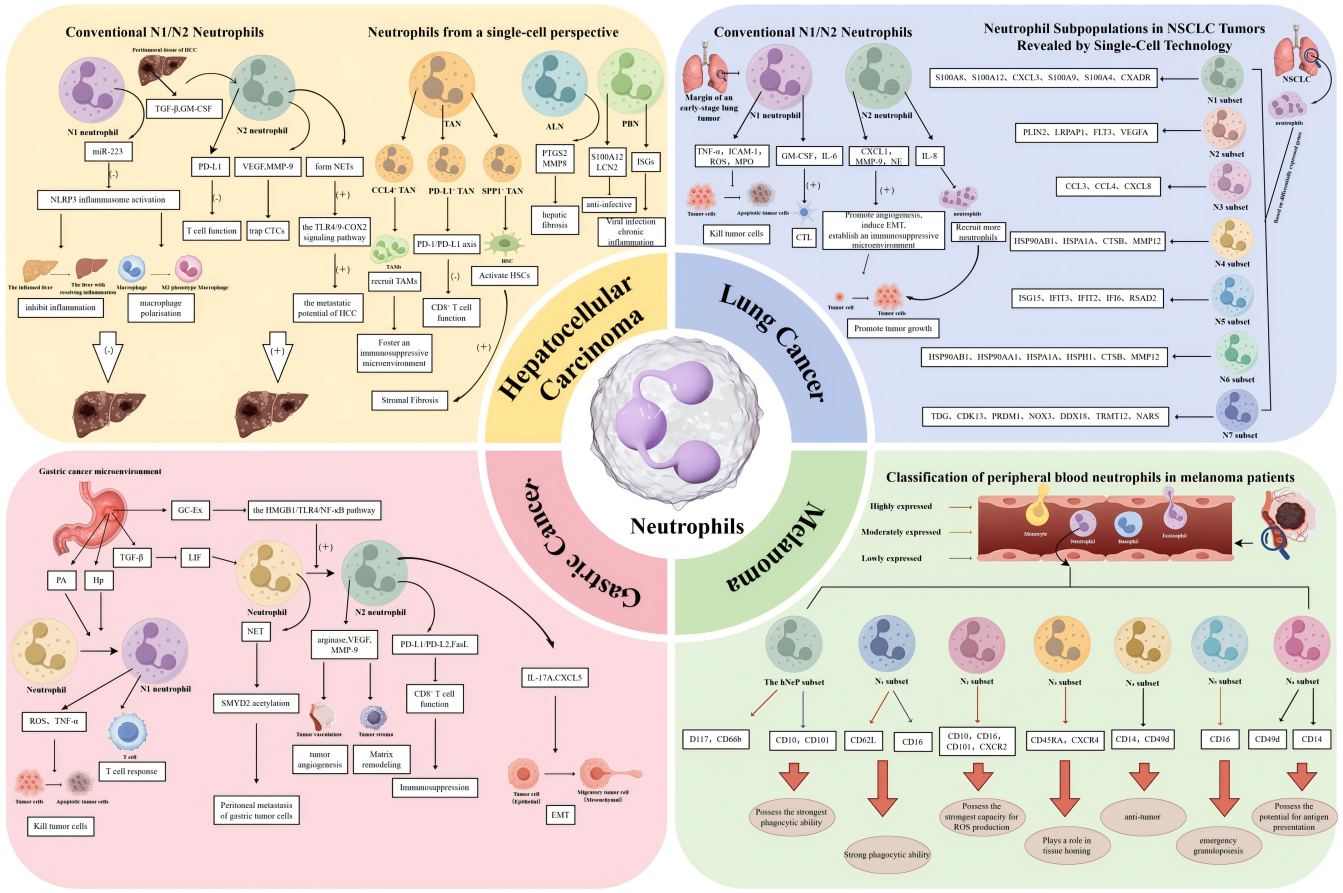
Functional heterogeneity, molecular mechanisms, and clinical relevance of neutrophils across four cancer types. This schematic delineates the distinct neutrophil subpopulations, their regulatory mechanisms, and dual functions within the TMEs of HCC, lung cancer, GC, and melanoma, as characterized by both traditional paradigms and single-cell high-resolution technologies. HCC is depicted in two contrasting paradigms. The left panel, representing the traditional N1/N2 Paradigm, illustrates the balance between anti-tumour N1 neutrophils, such as HDNs that generate ROS and miR-223, and pro-tumour N2 neutrophils, including LDNs that are stimulated by TGF-β and GM-CSF to express PD-L1 and form NETs. In contrast, the right panel, adopting a Single-Cell Perspective, reveals the heterogeneity of TANs, ALNs, and PBNs as elucidated by scRNA-seq. This analysis identifies pro-tumour subpopulations, including CCL4-positive TANs, which facilitate macrophage recruitment; PD-L1-positive TANs, which suppress T cell activity; and SPP1-positive TANs, which contribute to fibrosis. Lung Cancer. The left panel illustrates the traditional N1/N2 paradigm, depicting the polarization of TANs into anti-tumour N1 phenotypes, induced by IFN-β, and pro-tumour N2 phenotypes, induced by TGF-β. The right panel provides a single-cell perspective, identifying seven distinct neutrophil clusters (N1-N7) through scRNA-seq in NSCLC. This analysis emphasizes key clusters, including N1 (mature), N2 (pro-angiogenic), N3 (pro-inflammatory), and N5 (interferon-responsive). Gastric cancer is examined with a focus on the origin, polarization, and functions of TANs. The accompanying diagram illustrates the recruitment of TANs from the bone marrow and their subsequent polarization into N1 (anti-tumour) and N2 (pro-tumour) phenotypes, influenced by factors such as TGF-β and IL-17A. The diagram further emphasizes key pro-tumour mechanisms mediated by N2 TANs, including NETosis through the HMGB1/TLR4 pathway, exosome secretion of miR-4745-5p, the promotion of angiogenesis via VEGF, EMT, and the induction of immunosuppression, notably through the PD-L1 pathway. In the context of melanoma, circulating neutrophils in patients who have not yet undergone treatment are classified into seven distinct subsets through CyTOF analysis. These subsets include a hNeP and six additional subsets labelled N_1_ through N_6_. Each subset is characterized by specific surface markers, such as CD10, CD16, and CD101, which are associated with the stage of the disease and the functional capacities of the cells, including phagocytosis and ROS production. Arrows are utilized to denote the pathways of differentiation, recruitment, signalling, or functional effects.

Neutrophils are central components of innate immunity and are increasingly recognised as regulators of tumour progression. They provide new opportunities for therapeutic intervention, but their heterogeneity complicates both mechanistic understanding and clinical translation. This section synthesises current research on neutrophil heterogeneity within the TME, emphasising classification systems based on phenotypic characteristics and functional specialisation (**Figure 4**).

### 4.1 Neutrophils in hepatocellular carcinoma

HCC is one of the leading causes of cancer-related mortality worldwide, with its TIME exerting a major influence on progression and therapeutic response (135). Primary liver cancer (PLC) comprises three main histological subtypes: HCC, intrahepatic cholangiocarcinoma (ICC), and combined hepatocellular-cholangiocarcinoma (CHC) (136). Recent advances in single-cell RNA sequencing (scRNA-seq) have provided insight into the diversity of immune cell populations, including neutrophils, in liver cancer. Neutrophils in HCC demonstrate dual functionality: they can suppress tumour growth via antimicrobial and cytotoxic activity, yet also promote angiogenesis, immune suppression, and metastasis (38) (**Figure 4**).

#### 4.1.1 Neutrophil heterogeneity: from conventional perspectives to single-cell insights

##### 4.1.1.1 Functional subsets and mechanisms of conventional N1/N2 neutrophils

In HCC, neutrophils are commonly classified as antitumour N1 or protumour N2 subsets. N1 neutrophils exert tumouricidal effects through ROS release, cytotoxic granules, and activation of DCs and CD8^+^ T cells. They also secrete microRNA-223 (miR-223), which suppresses NOD-, LRR- and pyrin domain-containing protein 3​ (NLRP3) inflammasome activation, promotes resolution of hepatic inflammation, and drives macrophage polarisation towards a reparative type 2 macrophage (M2) phenotype (137). HDNs, identified by a CD16high/CD62Lhigh phenotype, represent a mature subset enriched in HCC patients with improved prognosis, with overall survival (OS) extended by approximately six months (p = 0.01) (21).

By contrast, N2 neutrophils promote HCC progression through secretion of pro-angiogenic factors (e.g., VEGF, MMP-9), immunosuppressive molecules (e.g., PD-L1, Arg-1), and NETs, which capture CTCs. He et al (138) reported that TGF-β and GM-CSF in peritumoural tissues induce PD-L1 upregulation on N2 neutrophils, suppressing T-cell activity. NETs also enhance HCC metastatic potential by activating Toll-like Receptor 4/9 (TLR4/9)-Cyclooxygenase-2 (COX2) signalling (139). LDNs, recruited via the CXCL5/CXCR2 axis, facilitate angiogenesis and immune evasion (140). LDNs further suppress T-cell proliferation by depleting arginine through Arg-1 secretion (141). The proportion of LDNs in peripheral blood correlates strongly with portal vein tumour thrombus incidence in HCC patients (r = 0.71, p < 0.001), highlighting their value as predictive biomarkers (139). Overall, N1 and N2 neutrophils exert diametrically opposed effects in HCC. Their balance may critically influence tumour progression, metastasis, and therapy responsiveness, forming the basis for neutrophil-targeted immunotherapeutic strategies.

##### 4.1.1.2 Functional subsets of neutrophils identified by single-cell sequencing

Neutrophils were once regarded as short-lived and homogeneous. However, recent studies have demonstrated marked heterogeneity within tumours. Using scRNA-seq, Zhang et al. (135) profiled 34,307 neutrophils from tumour tissue, adjacent non-tumour liver tissue, and peripheral blood of patients with HCC, identifying 11 functionally distinct subsets. These were grouped into three categories according to tissue origin and molecular features (135).

The first category comprises peripheral blood neutrophils (PBNs), such as Neu_02_s100 calcium binding protein A12 (S100A12) and Neu_03_interferon-stimulated gene 15 (ISG15), which express high levels of antimicrobial peptide genes (e.g., S100A12, Lipocalin 2 [LCN2]), consistent with anti-infective activity. The Neu_03_ISG15 subset additionally shows activation of interferon-stimulated genes (ISGs), potentially associated with viral infection or chronic inflammation (135). The second category includes adjacent liver tissue neutrophils (ALNs), exemplified by Neu_05_elongation factor for RNA polymerase II 2 (ELL2), which express matrix remodelling genes such as prostaglandin-endoperoxide synthase 2 (PTGS2) and matrix metalloproteinase 8 (MMP8). These neutrophils may contribute to hepatic fibrogenesis and tissue repair in cirrhosis (135, 141). The third category consists of TANs, including Neu_09_interferon-induced protein with tetratricopeptide repeats 1 (IFIT1), Neu_10_secreted phosphoprotein 1 (SPP1), and Neu_11_CCL4, which display strong pro-tumourigenic functions. Single-cell trajectory analysis suggests that TANs arise primarily through phenotypic remodelling of circulating neutrophils, rather than through local proliferation (135, 142). Cross-cancer comparative analyses further indicate that HCC TANs show unique metabolic programming, characterised by upregulation of glycolytic genes (HK2, PFKP) and fatty acid oxidation genes (carnitine palmitoyltransferase 1A[CPT1A]). Such metabolic reprogramming may be a central driver of their functional heterogeneity (143).

##### 4.1.1.3 Functional subsets of TANs and their mechanisms

TANs display substantial heterogeneity in the HCC microenvironment, with distinct subsets contributing to tumour progression and immune regulation through specialised mechanisms. CCL4^+^ TANs (Neu_11_CCL4) act as macrophage-recruiting “accomplices.” They express high levels of CCL3 and CCL4, which recruit TAMs via the CCL4-CCR5 axis, fostering an immunosuppressive niche (135). *Ex vivo* co-culture of hepatoma cells with neutrophils confirmed CCL4 upregulation, and patient-derived TANs secreted significantly higher levels of CCL4 than non-TANs (135).

PD-L1^+^ TANs (e.g., Neu_09_IFIT1) function as T-cell “suppressors.” By overexpressing PD-L1, they inhibit CD8^+^ T-cell cytotoxicity through the PD-1/PD-L1 pathway. CD8^+^ T cells co-cultured with PD-L1^+^ TANs exhibited markedly reduced IFNγ and granzyme B (GZMB) expression, an effect reversed by anti-PD-L1 antibodies (43). Assay for Transposase-Accessible Chromatin with high throughput sequencing (ATAC-seq) analysis further revealed increased chromatin accessibility of CD274 (encoding PD-L1) in TANs compared with non-TANs, suggesting epigenetic regulation of this phenotype (135).

SPP1^+^ TANs (Neu_10_SPP1) serve as stromal “facilitators.” By overexpressing osteopontin (SPP1) and integrin Integrin subunit beta 1 (ITGB1), they activate the PI3K-AKT pathway in hepatic stellate cells (HSC), driving stromal fibrosis and remodelling (135, 144). This subset is enriched in ICC, where SPP1^+^ TAN abundance correlates with serum carbohydrate antigen 19-9 (CA19-9) levels (r = 0.62, p = 0.003) and increased postoperative recurrence risk ([HR] = 1.8, p = 0.02), consistent with ICC’s poor prognosis (21, 135).

#### 4.1.2 Clinical significance and therapeutic potential of neutrophils in liver cancer

##### 4.1.2.1 Prognostic biomarkers

The Tumour-Immune Microenvironment Enhancement by Linking All Signals to Effector Responses (TIME-LASER) classification identified an immunosuppressive myeloid subtype enriched in TANs (Tumour Immune Microenvironment - Instruction, Suppression, Memory [TIME-ISM]), which correlated with shorter progression-free survival (PFS) (135). Specific TAN subsets (e.g., Neu_09, Neu_10, Neu_11) may therefore serve as independent prognostic indicators. Systemic markers also carry prognostic value: the NLR is a strong predictor of clinical outcomes. In pancreatic neuroendocrine tumours (pNETs), an NLR ≥ 4 was associated with liver metastasis and poor prognosis (145). In HCC, a high NLR (>3) correlated with advanced stage, vascular invasion, and reduced OS (146), likely reflecting suppression of lymphocyte-mediated antitumour responses by expanded neutrophil populations.

##### 4.1.2.2 Spatial distribution and functional heterogeneity of TANs

In HCC tissues, TANs are enriched at invasive margins and metastatic foci. scRNA-seq analyses identified two major TAN subsets: a pro-angiogenic subset (matrix metalloproteinase-9 positive, vascular endothelial growth factor A positive [MMP-9^+^VEGF-A^+^]) and an immunosuppressive subset (programmed death-ligand 1 positive, arginase 1 positive [PD-L1^+^ARG1^+^]). The latter contributes to immune evasion by inhibiting CD8^+^ T-cell activity (135). TAN density is higher in hepatic metastatic lesions than in primary tumours and correlates with complement pathway activation, suggesting that neutrophils may facilitate metastasis via complement-mediated mechanisms (145).

##### 4.1.2.3 Therapeutic targets

Targeting TAN-mediated immune regulation offers opportunities for combination therapy. Inhibition of complement signalling (e.g., with anti-complement component 5a [C5a] antibodies) suppresses pro-tumour neutrophil functions by disrupting C5a-driven neutrophil-tumour interactions (147). Blocking the CCL4-CCR5 axis reduces TAM recruitment, reversing immunosuppressive conditions (135). Synergistic effects are evident when neutrophil-directed interventions are combined with checkpoint blockade. Targeting PD-L1^+^ neutrophils enhances anti-PD-1 efficacy, whereas combining anti-PD-L1 therapy with TAN depletion (e.g., anti-Ly6G antibodies) leads to potent tumour suppression in preclinical models (135). Clinically, atezolizumab (anti-PD-L1) plus bevacizumab (anti-VEGF) significantly improved survival in advanced HCC, in part through dynamic modulation of TAN phenotypes (148). Importantly, cross-species studies confirm high conservation of key TAN subsets (e.g., CCL4^+^ and PD-L1^+^), strengthening the translational potential of preclinical findings (135). Collectively, these results support TANs as promising therapeutic targets, enabling multidimensional modulation of the myeloid cell network to enhance immunotherapy efficacy.

### 4.2 Neutrophils in lung cancer

Lung cancer has the highest global incidence and mortality among malignant tumours. NSCLC accounts for nearly 85% of cases, yet the 5-year survival rate remains as low as 16% (141). The heterogeneity of immune cells within the TME plays a pivotal role in disease progression. TANs, a central component of the TME, have become a focus of investigation because of their functional plasticity and phenotypic diversity. Conventionally, TANs have been divided into pro-tumoural N2 and antitumoural N1 phenotypes (43). More recently, scRNA-seq has revealed additional complexity within neutrophil subpopulations (142) (**Figure 4**).

#### 4.2.1 Heterogeneity of neutrophils: from traditional perspectives to single-cell insights

##### 4.2.1.1 Conventional classification: N1 and N2 phenotypes

Within the lung cancer microenvironment, TANs display marked phenotypic plasticity, primarily differentiating into N1 or N2 subsets. N1-polarized (N1-TANs) are characterised by high expression of TNF-α and ICAM-1. They exert direct tumouricidal activity through ROS and MPO release, or suppress metastasis by secreting CCL2 (43, 143). Their phenotype is induced by IFN-β, whereas TGF-β signalling inhibits their antitumour effects (45). In early-stage lung cancer, N1-TANs are mainly localised to the tumour periphery, where they secrete GM-CSF and IL-6 to activate cytotoxic T lymphocytes (CTLs), thereby mediating tumour suppression (21).

By contrast, N2-polarized (N2-TANs) are driven by TGF-β signalling and express CXCL1, MMP-9, and norepinephrine (NE). These molecules promote tumour progression by facilitating angiogenesis, inducing EMT, and generating an immunosuppressive niche (43, 149). Clinically, enrichment of N2-TANs is strongly associated with reduced OS in lung cancer (HR = 1.9, p < 0.01) (140). N2-TANs also reinforce neutrophil infiltration through IL-8 secretion, establishing a self-sustaining pro-tumour feedback loop (145).

The phenotypic distribution of TANs evolves dynamically. In early tumours, N1-TANs dominate at invasive margins, whereas in advanced disease, neutrophil phenotypes progressively shift towards N2, with greater infiltration into the tumour core (144). This spatiotemporal pattern reflects immune remodelling during tumour progression and highlights the therapeutic potential of stage-specific modulation of TAN polarisation.

##### 4.2.1.2 Subpopulation diversity revealed by single-cell technology

Conventional flow cytometry, constrained by limited marker panels, cannot fully resolve neutrophil heterogeneity. Shi et al. (142) used scRNA-seq to analyse tumour tissues from nine patients with advanced NSCLC, profiling 1,820 neutrophils. Clustering identified seven distinct subpopulations (N1-N7), which were functionally annotated based on differentially expressed genes (DEGs).

The N1 subset exhibits high expression of classical neutrophil marker genes (e.g., S100 calcium binding protein A8 [S100A8], S100 calcium binding protein A9 [S100A9], CXCR2) and represents mature neutrophils. Enriched in lung adenocarcinoma (LUAD), these cells suppress tumour progression through inflammatory modulation (146). Activation of the CXCR2 signalling pathway promotes neutrophil chemotaxis to inflammatory sites and enhances antitumour immunity (147). The N2 subset expresses perilipin 2 (PLIN2) (lipid droplet-associated protein), LDL receptor related protein associated protein 1​ (LRPAP1) (lipid metabolism regulation), and VEGF-A, consistent with a lipid metabolic and pro-angiogenic phenotype. It supports nutrient supply within the TME and promotes angiogenesis. Pseudotime analysis indicates an early differentiation stage, with a decline in gene expression during development. The N3 subset demonstrates high expression of chemoattractant genes (CCL3, CCL4, CXCL8), driving pro-inflammatory activity and immune cell recruitment. It is enriched in lung squamous cell carcinoma (LUSC) and correlates with poor prognosis (OS; HR = 2.1, p < 0.01). N3 recruits Tregs and TAMs, reinforcing an immunosuppressive microenvironment (148). Both N4 and N5 subsets express heat shock protein (HSP) family genes (Heat shock protein 90 alpha family class B member 1 [HSP90AB1], ​​heat shock protein family A [Hsp70] member 1A [HSPA1A]) and proteases (cathepsin B [CTSB], matrix metalloproteinase 12 [MMP12]), linking them to stress responses and antiviral defence. N4 is incompletely characterised but likely participates in proteostasis regulation. N5 expresses interferon-induced antiviral genes (ISG15, interferon induced protein with tetratricopeptide repeats 3 [IFIT3]), is more prevalent in LUAD, and is positioned at a mature differentiation stage, suggesting involvement in antiviral immunity and potential enhancement of antigen presentation (148). The N6 subset displays an intermediate expression profile between N4 and N5, with pseudotime trajectory analysis indicating shared developmental pathways with N4. It may act as a transitional population during neutrophil adaptation. The N7 subset, distinct from others, expresses DNA repair and RNA synthesis-related genes (X-ray repair cross complementing 5 [XRCC5], DEAD-box helicase 18 [DDX18], ​​PR/SET domain 1 [PRDM1]), implicating roles in genome maintenance and growth regulation. Pseudotime analysis places N7 at an early differentiation stage, potentially associated with tumour-specific heterogeneity and genome instability (146) (**Table 4**).

**Table 4:** Classification and functional characteristics of neutrophil subsets N1-N7.

Subset	Key DEGs	Functional characteristics	Clinical/prognostic association
N1	S100A8, S100A12, CXCL3, S100A9, S100A4, CXADR	Mature neutrophils; regulate inflammation via chemotaxis and adhesion	Enriched in LUAD; potentially antitumour
N2	PLIN2, LRPAP1, FLT3, VEGF-A	Lipid metabolism; promotes angiogenesis	Supports angiogenesis in TME
N3	CCL3, CCL4, CCL20, CCL3L1, CCL4L2, CXCL8, CXCL2	Chemokine-rich; recruits Tregs/TAMs; pro-inflammatory	Enriched in LUSC; poor OS (HR = 2.1)
N4	HSP90AB1, HSP90AA1, HSPA1A, HSPH1, CTSB, MMP12	Heat shock/stress response	Function unclear; overlaps with N6
N5	ISG15, IFIT3, IFIT2, IFI6, RSAD2	Interferon-induced antiviral response; possible antigen presentation	Enriched in LUAD; mature phenotype
N6	HSP90AB1, HSP90AA1, HSPA1A, HSPH1, CTSB, MMP12	Transitional subset; HSP regulation	Shared functions with N4, distinct trajectory
N7	TDG, CDK13, PRDM1, NOX3, DDX18, TRMT12, NARS	DNA repair, RNA synthesis, growth regulation	Early differentiation; tumour-specific role

Earlier classifications, often based on murine models or density-gradient separation (e.g., LDN vs. HDN) (150), underestimated this diversity. Zilionis et al. (151)identified five TAN subsets in lung cancer, where hN1 (mature) resembled Shi’s N1, and hN2 (interferon-responsive) paralleled N5. The discovery of N3 and N7 by Shi et al. (142) filled critical gaps, revealing pro-inflammatory and tumour-specific neutrophil populations previously uncharacterised.

#### 4.2.2 Developmental heterogeneity, functional plasticity, and clinical translation potential of TANs

TANs display complex developmental trajectories and functional heterogeneity in the lung cancer microenvironment. Pseudotime analysis reveals distinct differentiation lineages: N1 and N5 subsets represent terminally differentiated mature neutrophils, marked by progressive upregulation of signature genes (CXCR2, S100A8) and concurrent downregulation of MMP12 and PLIN2. In contrast, N7 is localised to early differentiation stages and likely retains immature or precursor-like properties. N4 and N6 share HSP-related signatures, suggesting roles in stress response and proteostasis regulation (141, 146). This developmental heterogeneity underlies TAN functional duality, with subsets exerting either pro-tumourigenic (e.g., N3, N2) or antitumourigenic (e.g., N1, N5) activity. Among the pro-tumourigenic subsets, N3 recruits immunosuppressive cells and activates VEGF-A signalling through high expression of chemoattractants (CCL20, CXCL8). Elevated expression of its signature genes (CCL20, ​​interleukin-1 receptor associated kinase 2 [IRAK2]) is significantly associated with reduced OS. N2-TANs secrete MMP-9 and NE to degrade the ECM and promote metastasis via NET formation, which activates the TLR4/COX2 pathway (141, 150, 152). In contrast, tumour-suppressive neutrophil subsets exhibit antitumour activity: N1-TANs induce tumour apoptosis through ADCC and TNF-related apoptosis-inducing ligand(TRAIL) signalling, whereas N5 is enriched in LUAD and defined by IFN response genes, implicating a specialised role in antitumour immunity (26, 141).

The functional plasticity of TANs is tightly associated with their interactions with other immune populations. Cell-cell communication analyses show reciprocal regulation with macrophages via the CXCL8-CXCR2 axis: macrophage-derived TGF-β and IL-10 polarise neutrophils towards the pro-tumourigenic N2 phenotype, whereas chemoattractants secreted by N3 reinforce this immunosuppressive network (43). These dynamics explain the phenotypic evolution of TANs across tumour stages, with early lesions dominated by N1-TANs at invasive margins and advanced tumours enriched in N2-TANs within the core (144).

Research on TAN heterogeneity provides novel insights into lung cancer diagnosis and treatment. For prognosis, systemic inflammatory indices have predictive value: a high NLR (>5) or platelet-to-lymphocyte ratio (PLR >200) correlates with poor immunotherapy response (e.g., to nivolumab), reflecting neutrophil-derived immunosuppressive factors (IL-10, TGF-β) and possible MDSC infiltration (153). Conversely, patients with NLR <3 generally show better outcomes. Marker genes such as CCL20 and IRAK2 from the N3 subset also hold promise as liquid biopsy-based prognostic indicators (141, 154).

Therapeutic interventions targeting TANs have diversified: (1)Phenotype conversion: Galunisertib (a TGF-β receptor inhibitor) combined with atezolizumab (anti-PD-L1) reduced tumour volume by 62% (p < 0.001) by reversing N2 polarisation towards an N1 phenotype (142). (2)Chemotactic blockade: In a phase II trial, the CXCR2 inhibitor AZD5069 prolonged PFS by inhibiting neutrophil recruitment (155). (3)NET disruption: In murine models, Deoxyribonuclease (DNase) I reduced pulmonary metastatic nodules by 58% (p < 0.01); a clinical trial is ongoing (NCT03817320) (156). Importantly, the clinical implications of TANs appear subtype-specific. In LUSC, CD66b^+^ TANs correlate with favourable prognosis, whereas in LUAD, they associate with poor outcomes (142, 154). This contrast highlights the importance of precise subtype stratification for TAN-targeted therapies and suggests that markers such as PLR may carry differential prognostic value across histological subtypes, warranting further refinement.

### 4.3 Neutrophils in gastric cancer

GC is one of the most prevalent and lethal malignancies worldwide. Its development and progression are closely associated with the dynamic regulation of TANs within the TME. As key immune regulators, TANs display dual functionality: N1 neutrophils exert antitumour activity through cytotoxicity and immune activation, whereas N2 neutrophils promote invasion, angiogenesis, and immune evasion (156) (**Figure 4**).

#### 4.3.1 Origin and phenotypic characteristics of TAN subtypes

TANs derive from HSCs in the bone marrow, differentiate via GMPs, and migrate into the TME through CXCR2- and CXCR4-dependent chemotaxis (157–159). Their polarisation is tightly regulated by cytokines such as TGF-β, IL-17A, and IFN-γ, as well as local signalling pathways. N1-TANs (CD16^+^) exhibit pronounced antitumour activity, releasing ROS and TNF-α to directly kill tumour cells, whereas also promoting T-cell responses through antigen presentation (158–160). In contrast, N2-TANs (CD66b^+^) adopt a protumour phenotype, characterised by secretion of Arg-1, MMP-9, and VEGF, thereby driving angiogenesis, ECM remodelling, and immune suppression (159–162). Recent findings suggest that TANs may not conform to a strict N1/N2 dichotomy but rather exist along a functional continuum. PMN-MDSCs overlap phenotypically and functionally with N2-TANs, though their markers and regulatory pathways remain incompletely defined (36, 160, 162). This continuum highlights the dynamic plasticity of TANs and their context-dependent roles at different stages of GC progression. Clinically, an elevated NLR is associated with poor prognosis in GC, reflecting systemic inflammation and immunosuppression (160, 162).

#### 4.3.2 Molecular mechanisms regulating TAN polarisation

Several pathways modulate TAN polarisation in GC. Phosphatidic acid (PA) promotes N1 polarisation by activating Hippo signalling pathway (Hippo)-Yes-associated protein (YAP) signalling, thereby enhancing tumouricidal activity (163). Expression of Transducer of ERBB2, 1 (TOB1 in CD66b^+^ neutrophils correlates positively with patient survival and supports N1 polarisation. Conversely, gastric cancer cell-derived exosomes (GC-Exs) induce N2 polarisation through the HMGB1/TLR4/nuclear factor kappa-light-chain-enhancer of activated B cells (NF-κB) pathway, promoting metastasis (164). TGF-β drives neutrophils to release NETs via the Smad2/3-leukaemia inhibitory factor (LIF) axis, accelerating peritoneal dissemination (165). Helicobacter pylori infection can transiently induce N1-like responses, but chronic inflammation caused by persistent infection increases carcinogenic potential (166, 167).

#### 4.3.3 Dual roles of TAN subsets in GC pathogenesis

TAN subsets contribute to GC pathogenesis via distinct mechanisms. N1 TANs eliminate tumour cells by releasing ROS and TNF-α and activating CD8^+^ T-cell immunity. However, excessive ROS may exacerbate genotoxic stress and chronic inflammation, increasing carcinogenic potential (168). N2 TANs drive GC progression through multiple pathways. NETs upregulate N-acetyltransferase 10 (NAT10)-mediated SET and MYND domain-containing 2 (SMYD2) acetylation, enhancing invasion (168), whereas TAN-derived exosomal microRNA 4745-5p (miR-4745-5p) suppresses slit guidance ligand 2 (SLIT2) expression to facilitate metastasis (169). N2 TANs also secrete VEGF and MMP-9 to stimulate angiogenesis, a process reinforced by NET-mediated activation of platelet-endothelial adhesion molecules (170–172). Hypoxia-induced NETs, via the HMGB1-TLR4/p38 mitogen-activated protein kinase (p38 MAPK) axis, create a pro-angiogenic feedback loop (173).

TANs further regulate metastasis by promoting EMT. Family with sequence similarity 3, member C (FAM3C) activates c-Jun N-terminal kinase (JNK)-zinc finger E-box binding homeobox 1 (ZEB1)/Snail family transcriptional repressor 1 (Snail signalling) (174), whereas IL-17A and CXCL5 induce EMT through JAK2/signal transducer and activator of transcription 3 (STAT3) and extracellular signal-regulated kinase (ERK)/p38 pathways, respectively. Neutralisation of IL-6/interleukin-23 (IL-23) partially reverses EMT (175, 176). Immunosuppression is also reinforced by PD-L1, programmed death-ligand 2 (PD-L2), and FasL expression on N2 TANs, which inhibit CD8^+^ T-cell function (177, 178). Triggering receptor expressed on myeloid cells 1 (TREM1) signalling promotes macrophage polarisation via NET formation, exacerbating the immunosuppressive TME (179). Importantly, IFIT1^+^ TANs contribute to ICI resistance by upregulating PD-L1 and inducing EMT (180), whereas interleukin-17 (IL-17)-activated neutrophils are associated with PD-1 therapy resistance (181). These findings untangle the role of TANs in GC therapeutic resistance and establish a mechanistic foundation for combination therapies targeting specific neutrophil subsets.

### 4.4 Neutrophil heterogeneity in melanoma

As central effectors of innate immunity, neutrophils have gained increasing attention for their functional heterogeneity in cancer. Using mass cytometry (CyTOF) and flow cytometry, Zhu et al. (182) characterised circulating neutrophil subsets in treatment-naïve melanoma patients and correlated their distribution with disease stage. Seven functionally distinct populations were identified, differing markedly in phagocytic activity and ROS production, thereby highlighting the dual roles of neutrophils in melanoma progression (**Figure 4**).

#### 4.4.1 Neutrophil subtypes in melanoma

Conventional flow cytometry is limited by restricted marker panels, whereas CyTOF allows broader phenotypic resolution. Applying a 40-marker CyTOF panel, Zhu et al. (182) identified one precursor population (hNeP) and six novel subsets (N_1_-N_6_). The immature precursor subset hNeP (CD117^+^CD66b^+^), lacking maturation markers CD10 and CD101, exhibited the strongest phagocytic capacity, comparable to monocytes, and was significantly expanded in advanced melanoma, suggesting an association with tumour progression (135, 141). The N_1_ subset (CD16dimCD62Lbright), characterised by low CD16 and high CD62L expression, aligned with a “banded” neutrophil phenotype. It retained strong phagocytosis but showed weaker ROS production (145). The terminally mature N_2_ subset (CD10^+^CD16brightCD101^+^), marked by high CXCR2 expression, generated the strongest ROS response; however, its frequency declined in advanced disease, indicating depletion of mature neutrophils in an inhospitable tumour milieu (135, 140). The N_3_ subset (CD45RA^+^ CXCR4^+^), resembling a senescent phenotype, displayed weak baseline phagocytosis but strong ROS induction, possibly reflecting tissue homing functions (45). The N_4_ subset (CD14^+^CD49d^+^), associated with antitumour activity, showed the lowest ROS levels, suggesting suppression within the TME (147). The N_5_ subset (CD10^-^CD16^+^CD34^-^), a non-proliferative immature population with low phagocytic activity, was markedly expanded in melanoma patients, potentially reflecting “emergency granulopoiesis” (155). Finally, the N_6_ subset (CD14^+^CD49d^+^CD101^-^), similar to N_4_ but lacking CD101, expressed CD14 and may possess antigen-presenting potential, with intermediate functional characteristics (147).

These subsets overlap with previously reported populations. CD10^+^ neutrophils (N_2_) share immunosuppressive properties with those described by Marini et al. (183), whereas CD49d^+^ precursors (N_3_, N_6_) resemble the preNeu subset identified by Evrard et al. (7) in mice. The CD14^+^ subset (N_6_) parallels tumour-suppressive neutrophils reported by Singhal et al. (184). However, variability in marker usage across studies highlights the need for standardised classification criteria (185).

#### 4.4.2 Clinical relevance of neutrophil subsets

Immature subsets correlate strongly with melanoma progression. The combined proportion of hNeP, N_1_, and N_3_ increased from <10% in early-stage patients to ~40% in advanced-stage cases, whereas mature N_2_ subsets declined. Regression analyses showed a negative association between N_2_ frequency and disease stage, consistent with tumour-driven “emergency granulopoiesis” (182, 186). Visualization of t-distributed stochastic neighbour embedding (viSNE) clustering stratified patients into four groups (A-D). Late-stage patients were concentrated in groups C and D, characterised by high immature subset abundance, whereas early-stage patients displayed predominance of mature N_2_ neutrophils. These findings suggest that neutrophil heterogeneity patterns could serve as prognostic biomarkers, although larger clinical validation is needed.

#### 4.4.3 Functional heterogeneity of neutrophil subsets: the dual-edged sword of phagocytosis and ROS production

Neutrophil subsets display striking functional heterogeneity in both phagocytic capacity and ROS generation, thereby shaping the balance of the TIME. In terms of phagocytosis, immature precursors such as hNeP and the N1 subset demonstrate strong activity comparable to monocytes, whereas the more differentiated N2 and N5 subsets are less efficient on a per-cell basis. Despite this lower efficiency, the predominance of N2 and N5 in peripheral circulation contributes to roughly 50% of total phagocytic activity in patients, indicating an important role in tumour- associated antigen processing and presentation (182). This gradient in phagocytic function may influence the clearance of tumour antigens and the subsequent priming of antigen-specific T cells, ultimately modulating the strength of adaptive antitumour immunity.

ROS regulation adds further complexity. Subsets such as N2 and N5 exhibit the highest ROS production capacity, whereas N4 and N6 generate the lowest levels. ROS themselves exert concentration-dependent effects within the tumour milieu: physiological levels support T cell activation and proliferation, but excessive ROS induce lymphocyte apoptosis and immune suppression. High ROS production in N2 may therefore drive immune evasion by impairing T cell function, whereas moderate ROS release from hNeP may help preserve homeostasis in the immune microenvironment (182). These functional divergences are highlighted by therapeutic studies. ROS inhibitors suppressed the activity of N2 neutrophils by 85%, compared with only 25% inhibition in N4, highlighting the presence of distinct regulatory networks across subsets. Collectively, these findings reveal a finely tuned division of labour among neutrophil subsets in tumour immune regulation and establish a mechanistic foundation for the development of immunotherapies aimed at selectively targeting defined subsets.

### 4.5 Cytokine-mediated regulatory mechanisms of neutrophils in tumours and their pro-tumour effects

Neutrophils play a pivotal role in tumour progression, with their activity tightly regulated by a complex network of cytokines. Key mediators such as CXCL5, IL-6, and TGF-β activate signalling cascades, including CXCR2/PI3K/Akt, STAT3, and Smad pathways, that drive neutrophil recruitment and polarisation towards the pro-tumourigenic N2 phenotype, thereby generating TANs. These TANs exert pro-angiogenic functions through VEGF/MMP-9 release, facilitate EMT via the Glycogen synthase kinase-3 beta (GSK-3β)/Snail pathway, and suppress adaptive immunity through ARG1 and PD-L1 expression. Distinct tumour types exhibit context-dependent cytokine-neutrophil interactions. In HCC and BC, CXCL5 activates the CXCR2/PI3K/Akt/GSK-3β/Snail pathway, driving EMT and metastasis (187), whereas IL-6 induces N2 polarisation via STAT3 signalling, enhancing VEGF/MMP-9 release and angiogenesis (188). TGF-β prolongs neutrophil survival by inhibiting IFN-β signalling and promotes secretion of pro-metastatic factors such as MMP-9 (189). GM-CSF stimulates neutrophils to release oncostatin M (OSM), facilitating tumour cell detachment from the primary lesion (190). IL-17 induces G-CSF-dependent neutrophil expansion via γδ T cells, suppressing CD8^+^ T-cell activity and promoting metastasis (191). G-CSF itself upregulates expression of the pro-angiogenic factor Bv8, mobilising neutrophils to lung tissue and fostering pre-metastatic niche formation (192).

In NSCLC, LTB4 recruits neutrophils through the leukotriene B4 receptor-1(BLT1) receptor, activating a ROS-dependent DNA damage pathway (193), whereas IL-8 promotes neutrophil infiltration via the CXCR1/CXCR2 axis, leading to MMP-9 release, ECM degradation, and angiogenesis (194). In CRC, CXCL2-mediated neutrophil recruitment drives ROS release and epithelial DNA damage (195), whereas IL-17 promotes accumulation of immunosuppressive neutrophils through the γδ T cell-CCL2/CCL20 axis (196). In head and HNSCC, pancreatic Cancer (PDA), and glioma, macrophage migration inhibitory factor (MIF) enhances tumour cell migration by upregulating ICAM-1 (197). NETs represent a critical pro-tumour mechanism. They physically capture CTCs to promote liver metastasis (150). IL-17 can induce NETosis and N2 polarisation by activating the complement component 3a (C3a) receptor, thereby fostering thrombosis (198). In glioma, IL-17 promotes blood-brain barrier penetration via LFA-1 integrin, accompanied by IL-17 and NET release, which exacerbate neuroinflammation and tumour progression (199). Collectively, these mechanisms highlight the dynamic cytokine-driven regulation of neutrophils in the TME. Their phenotypic plasticity, particularly the N1/N2 transition, highlights potential therapeutic targets. Future studies should clarify the spatiotemporal dynamics of cytokine-mediated neutrophil heterogeneity across tumour types and dissect their cooperative crosstalk with other immune cells (**Table 5**)

**Table 5:** Cytokine-mediated regulation of neutrophils in tumours.

Tumour type	Key cytokine	Major function/mechanism	Reference
HCC	CXCL5	Activates PI3K/Akt/GSK-3β/Snail pathway via CXCR2, promoting EMT and metastasis	(187)
	IL-6	Induces N2 polarisation via STAT3 signalling, enhances VEGF/MMP-9 release, drives angiogenesis	(188)
	TGF-β	Suppresses IFN-β signalling, prolongs neutrophil survival, promotes MMP-9 secretion	(189)
Breast Cancer	GM-CSF	Induces neutrophil OSM release, facilitating tumour cell detachment	(190)
	IL-17	Promotes G-CSF-dependent neutrophil expansion via γδ T cells, suppresses CD8^+^ T cells, drives metastasis	(191)
	G-CSF	Upregulates pro-angiogenic Bv8, mobilises neutrophils to lung pre-metastatic niches	(192)
	CXCL8	Attracts VEGFR1^+^ neutrophils via CXCR2, supporting lung metastasis	(200)
NSCLC	TGF-β	Induces N2 polarisation via Smad, promotes ARG1/PD-L1 expression, suppresses T cells	(43)
	LTB4	Recruits neutrophils via BLT1, activates ROS-dependent DNA damage	(193)
	IL-8	Recruits neutrophils via CXCR1/CXCR2, promotes ECM degradation and angiogenesis	(194)
CRC	IL-1β	Activates IL-6/STAT3 pathway, promoting inflammation-associated tumourigenesis	(201)
	CXCL2	Recruits neutrophils to release ROS, causing epithelial DNA damage	(195)
	IL-17	Recruits immunosuppressive neutrophils via γδ T cell-CCL2/CCL20 signalling	(196)
HNSCC	MIF	Upregulates ICAM-1, enhancing tumour cell migration	(197)
	IL-6	Prolongs neutrophil survival via STAT3, promotes secretion of pro-inflammatory mediators (e.g., IL-8)	(202)
PDA	NETs	Capture CTCs, promote liver metastasis	(150)
	IL-17	Activates C3a receptor, induces NETosis and N2 polarisation, promotes thrombosis	(198)
Glioma	IL-17	Facilitates blood-brain barrier penetration via LFA-1, releases IL-17/NETs, exacerbates neuroinflammation	(199)
	NETs	Directly participate in neuroinflammatory responses	(199)

## 5 Spatial omics analysis of tumour-associated neutrophils

Advancements in spatial transcriptomics and single-cell multi-omics have transformed the study of tumour-associated neutrophils (TANs) from broad phenotypic descriptions to a focus on their spatiotemporal dynamics and microenvironmental organization. Ng et al., using intravital multiphoton imaging and spatial transcriptomic analysis, revealed the “deterministic reprogramming” of TANs in tumours, driven by local signal gradients like IL-1β and G-CSF (170). At the tumour’s invasive front, TANs enhance factors such as MMP9 and VEGFA via STAT3 signalling to aid invasion and metastasis. Conversely, in the necrotic core, they express immunosuppressive molecules like ARG1, PD-L1, and IDO1, creating an immune-privileged environment that inhibits CD8^+^ T cell activity (43, 170). The discovery of spatial functional heterogeneity reveals why traditional bulk sequencing cannot accurately determine the clinical impact of TANs, as their pro-tumour or anti-tumour effects rely heavily on their tumour location and interactions with nearby cells. Research shows that TANs’ distribution aligns with tumour progression. In early stages, neutrophils can trigger tumour cell death via the TRAIL/FasL pathway or through ADCC (203). As tumours advance, the microenvironment, influenced by TGF-β, G-CSF, and IL-1β, shifts neutrophils to a pro-tumour state, forming N2-like TANs or PMN-MDSCs (161). Neutrophils also release NETs, aiding tumour spread and activating CAFs via TLR9, creating a supportive tumour environment (204, 205).

The main challenge in current therapeutic strategies is achieving precise spatial targeting. Future research should adopt multidisciplinary approaches to address this issue in tumour-associated neutrophil (TAN) studies. Technically, integrating multi-omics spatial atlases with dynamic *in vivo* imaging is crucial for mapping TAN interactions with stromal cells and understanding niche-specific signalling dynamics (206, 207). Additionally, developing computational models and AI algorithms to analyse spatial data can help predict neutrophil behaviour and identify key intervention targets (208). In the context of therapeutic translation, it is imperative to focus efforts on the development of advanced delivery systems that are responsive to the TME. These systems, such as antibody-drug conjugates (ADCs) or nanocarriers, are designed to recognize the biochemical characteristics of specific tumour regions, including hypoxia, distinct pH levels, or enzymatic activity. This approach facilitates the targeted elimination of tumour-promoting neutrophil subsets, such as LOX-1^+^ PMN-MDSCs, while preserving or activating anti-tumour neutrophil subsets, such as ICAM1^+^ CD62Llow cells (61, 209). Additionally, the integration of strategies targeting neutrophil recruitment pathways, such as CXCR1/2 inhibitors, with immune checkpoint blockade, shows potential in reversing the immunosuppressive microenvironment. This is achieved by modulating the spatiotemporal distribution of tumour-associated neutrophils (TANs), thereby enhancing the efficacy of existing therapies (210). It is essential to underscore that the realization of these objectives necessitates the urgent development of more precise preclinical models. These models, such as organoid systems or humanized animal models, must be capable of replicating the spatial heterogeneity of human tumours, thereby providing a robust platform for the validation of these targeting strategies.

Understanding the spatiotemporal regulation of TANs is crucial for tumour immunotherapy. Spatial multi-omics helps clarify their roles, enhancing our grasp of tumour diversity and shifting research towards predictive science. Future interdisciplinary efforts combining spatial biology, engineering, and immunotherapy aim to target TAN heterogeneity precisely, potentially leading to new treatments for inflammation-related diseases (170, 210).

## 6 Conclusion and perspectives

Neutrophils, as pivotal effector cells of innate immunity, display remarkable functional plasticity and phenotypic heterogeneity within the TME. Traditionally regarded as antimicrobial effectors, they are now recognised as direct regulators of tumour progression. Distinct subsets, including HDNs, LDNs, and PMN-MDSCs, exert dual antitumour or protumour effects. Investigation of neutrophil functions within tumours has therefore become a central research focus, particularly in view of drug resistance encountered with conventional T cell-based therapies. Earlier studies highlighted neutrophil-derived responses such as oxidative stress, NETs, and cytokine-mediated regulation. Although some of these mediators are also secreted by other immune or tumour cells, substances such as MMP-9 are predominantly of neutrophil origin. This distinction highlights the need for comparative studies and experimental evidence to establish neutrophil-based therapies as complementary or alternative strategies to T cell-based treatments, with the goal of identifying more effective clinical interventions.

Although reducing neutrophil numbers may be beneficial in tumours enriched with protumour subsets, such depletion also eliminates antitumour neutrophils (72). Therapeutic strategies should therefore be designed with pre-emptive measures to mitigate adverse effects. Targeting tumour surface molecules or cytokines that regulate neutrophil activity represents a rational approach to overcoming clinical drug resistance, suppressing protumour neutrophil functions, and synergising with novel immunotherapies to enhance efficacy.

Future research may focus on associations between HNA polymorphisms and tumour susceptibility or prognosis. Large-scale genome-wide association studies (GWAS) and retrospective clinical analyses could clarify links between specific HNA alleles (e.g., HNA-1, HNA-3, HNA-5) and tumour incidence, metastatic risk, or survival. Integration of scRNA-seq and spatial transcriptomics could further unravel how HNA polymorphisms influence neutrophil polarisation (N1 antitumour vs N2 protumour) in the TME. Moreover, therapeutic strategies such as HNA-targeting monoclonal antibodies—for example, against HNA-1a—could activate ADCC to eliminate tumour cells. Continued investigation of TANs as diagnostic and prognostic biomarkers is also warranted.

Current research faces limitations, including inconsistent classification criteria for neutrophil subsets, incomplete understanding of dynamic transitions, and underexplored spatiotemporal heterogeneity, metabolic reprogramming, and cross-disease features. Small sample sizes constrain generalisability, highlighting the need for expanded cohorts to validate classifications and regulatory mechanisms. Advancing neutrophil research from descriptive to mechanistic and translational domains will require interdisciplinary collaboration. Neutrophil-targeted therapies remain at an exploratory stage, with challenges in safety and specificity. Future research should aim to enhance AI algorithms for predicting neutrophil behaviour in specific microenvironments, improve microenvironment-responsive delivery systems, and develop precise preclinical models for accurate spatial targeting. In parallel, development of liquid biopsy biomarkers may further strengthen clinical applications. This field not only deepens understanding of tumour immune landscapes but also provides novel perspectives on challenges such as resistance to cancer immunotherapy and immune dysregulation in sepsis, highlighting its scientific and translational significance.

## Glossary

PMNs: polymorphonuclear neutrophils
HNA: the human neutrophil antigen
HSCs: Haematopoietic stem cells
HSC: hepatic stellate cells
GMP: granulocyte-macrophage progenitors
LDNs: low-density neutrophils
HDNs: high-density neutrophils
LD: low-density
PBMCs: peripheral blood mononuclear cells
NE: neutrophil elastase
SLE: systemic lupus erythematosus
BC: breast cancer
iLDN: immature LDN
VEGF: vascular endothelial growth factor
MPO: myeloperoxidase
NLR: neutrophil-to-lymphocyte ratio
NO: nitric oxide
PD-1/PD-L1: programmed cell death protein 1/programmed death-ligand 1
EMT: transition epithelial-mesenchymal transition
TANs: tumour-associated neutrophil
MDSCs: myeloid-derived suppressor cells
M-MDSCs: monocytic myeloid-derived suppressor cells
PMN-MDSCs: polymorphonuclear myeloid-derived suppressor cells
NADPH: nicotinamide adenine dinucleotide phosphate
ROS: reactive oxygen species
RNS: reactive nitrogen species
NETs: neutrophil extracellular traps
ECM: extracellular matrix
MMPs: matrix metalloproteinases
CTCs: circulating tumour cells
ICB: immune checkpoint blockade
ICAM1: intercellular adhesion molecule 1
TNF: tumour necrosis factor
TGF: transforming growth factor
GM-CSF: granulocyte-macrophage colony-stimulating factor
G-CSF: granulocyte colony-stimulating factor
PR: proteinase
CitH3: citrullinated histone H3
ADCC: antibody-dependent cellular cytotoxicity
TME: tumour microenvironment
FasR: fas receptor
FasL: fas ligand
4-HNE: 4-hydroxynonenal
HGF: hepatocyte growth factor
PRRs: pattern recognition receptors
TLR: toll-like receptor
MZ: marginal zone
BAFF: B cell-activating factor
APRIL: a proliferation-inducing ligand
APCs: antigen-presenting cells
MHC II: MHC class II
NK: natural killer
OVs: oncolytic viruses
Adv: adenovirus
HSV: herpes simplex virus
MV: measles virus
VACV: vaccinia virus
MVA: modified vaccinia ankara
VSV: vesicular stomatitis virus
PAD4: peptidylarginine deiminase 4
p-EMT: partial epithelial-mesenchymal transition
HMGB1: high-mobility group box 1
LTB4: leukotriene B4
BMP2: bone morphogenetic protein 2
PI3K/Akt: phosphoinositide 3-kinase/protein kinase B
MCAM/MUC18: melanoma cell adhesion molecule/melanoma-associated cell adhesion molecule (CD146)
ICI: checkpoint inhibitor
NSCLC: non-small cell lung cancer
CAF: cancer-associated fibroblast
GPI: glycosylphosphatidylinositol
SNPs: single-nucleotide polymorphisms
PNH: paroxysmal nocturnal haemoglobinuria
NAIN: neonatal alloimmune neutropenia
AIN: autoimmune neutropenia
TRALI: transfusion-related acute lung injury
MDS: myelodysplastic syndromes
CML: chronic myeloid leukaemia
AML: acute myeloid leukaemia
ANCA: anti-neutrophil cytoplasmic antibody
MPN: myeloproliferative neoplasm
PV: Polycythaemia vera
mPR3: membrane-bound proteinase 3
MPD: myeloproliferative disorder
uPAR: urokinase plasminogen activator receptor
CTL2: choline transporter-like protein 2
ARDS: acute respiratory distress syndrome
HSCT: haematopoietic stem cell transplantation
DCs: dendritic cells
ALNs: adjacent liver tissue neutrophils
ATAC-seq: assay for Transposase-Accessible Chromatin with high throughput sequencing
BLT1: leukotriene B4 receptor-1
CHC: combined hepatocellular-cholangiocarcinoma
CPT1A: carnitine palmitoyltransferase 1A
CTLs: cytotoxic T lymphocytes
CXCR: C-X-C motif chemokine receptor
CyTOF: mass cytometry
hNeP: neutrophil precursor
DEGs: differentially expressed genes
GC: gastric cancer
GC-Exs: gastric cancer cell-derived exosomes
GZMB: granzyme B
HCC: hepatocellular carcinoma
HNSCC: neck squamous cell carcinoma
HR: hazard ratio
HSP: heat shock protein
ICC: intrahepatic cholangiocarcinoma
IFIT1: interferon-induced protein with tetratricopeptide repeats 1
IFN: interferon
ISGs: interferon-stimulated genes
LIF: leukaemia inhibitory factor
LUAD: lung adenocarcinoma
LUSC: lung squamous cell carcinoma
MIF: macrophage migration inhibitory factor
N1-TANs: N1-polarized tumour-Associated Neutrophils
N2-TANs: N2-polarized tumour-Associated Neutrophils
OS: overall survival
OSM: oncostatin M
PA: phosphatidic acid
PBNs: peripheral blood neutrophils
PDA: pancreatic Cancer
PFS: progression-free survival
PLC: primary liver cancer
PLR: platelet-to-lymphocyte ratio
SPP1: secreted phosphoprotein 1 (Osteopontin)
STAT3: signal transducer and activator of transcription 3
TIME: tumour immune microenvironment
TRAIL: TNF-related apoptosis-inducing ligand
Tregs: regulatory T cells.
