# Supplementary material for: Mechanistic roles of neutrophil heterogeneity in tumour pathogenesis
Source: Front Immunol. 2025 Dec 10;16:1721090. doi: 10.3389/fimmu.2025.1721090 (PMC12727949; doi:10.3389/fimmu.2025.1721090)
Supplement: Supplementary file 1 [file Table1.docx]

**Table X Selected index of key terminology**

| Term | Description | Relationship with Neutrophils |
| --- | --- | --- |
| Key Cells | | |
| TANs | Neutrophils present within tumours and surrounding tissues that play complex and multifaceted roles in tumour initiation, progression, metastasis, and treatment response. | — |
| MDSCs | A population of immature myeloid cells with immunosuppressive functions under pathological conditions (e.g., cancer, chronic infections). They can be classified into monocytic MDSCs (M-MDSCs, phenotype: CD14^+^HLA^-^DR^-^/lo) and granulocytic MDSCs (G-MDSCs/PMN-MDSCs, phenotype: CD14^-^CD11b^+^CD33^+^CD15^+^/CD66b^+^), among other subsets. | PMN-MDSCs share overlapping features with neutrophils. Studies indicate that MDSCs can differentiate into granulocytes under *in vitro* conditions. |
| Substances Produced by Neutrophils | | |
| ROS/RNS | Reactive oxygen/nitrogen species that contribute to antimicrobial defence, antitumour immunity, and immunomodulation, but may also cause tissue damage and inflammatory responses under certain conditions. | Produced by neutrophils through multiple mechanisms including the nicotinamide adenine dinucleotide phosphate (NADPH) oxidase system, mitochondrial pathways, and nitric oxide synthase. |
| NET/NETs | Web-like structures composed of DNA, histones, and granular proteins that function in pathogen trapping, inflammatory responses, thrombosis, and tumour metastasis. | Released by neutrophils upon disease-related stimulation as fibrous extracellular structures. |
| Matrix-Degrading Enzymes | Enzymes capable of degrading ECM, including MMPs, serine proteases, and cysteine proteases. Among these, MMPs are the most extensively studied and functionally diverse. | Produced by various cell types, including neutrophils. |
| MMPs | A family of zinc-dependent proteases expressed in multiple cell types, with pivotal roles in NET formation and functional regulation. |  |
| Special Tumour Components | | |
| CTCs | Tumour cells shed from primary or metastatic sites into the bloodstream, serving as “seeds” for metastasis. They are important biomarkers for cancer diagnosis, prognosis, and treatment monitoring. | Exhibit dynamic interactions with neutrophils.. |
| Therapeutic Approaches | | |
| ICB | A cancer immunotherapy strategy that enhances antitumour immune responses by inhibiting immunosuppressive signalling pathways. | May synergise with neutrophil-targeted strategies for improved therapeutic efficacy. |
